# Development and exploitation of a novel mutant androgen receptor modelling strategy to identify new targets for advanced prostate cancer therapy

**DOI:** 10.18632/oncotarget.4347

**Published:** 2015-07-20

**Authors:** Daniel O'Neill, Dominic Jones, Mark Wade, James Grey, Sirintra Nakjang, Wenrui Guo, David Cork, Barry R. Davies, Steve R. Wedge, Craig N. Robson, Luke Gaughan

**Affiliations:** ^1^ Northern Institute for Cancer Research, Newcastle University, Newcastle Upon Tyne, NE2 4HH, UK; ^2^ AstraZeneca, Innovative Medicines, Discovery Sciences, Cambridge Science Park, Cambridge, CB4 0WG, UK

**Keywords:** prostate cancer, androgen receptor, mutation, anti-androgen-resistance, SGK1

## Abstract

The persistence of androgen receptor (AR) signalling in castrate-resistant prostate cancer (CRPC) highlights the unmet clinical need for the development of more effective AR targeting therapies. A key mechanism of therapy-resistance is by selection of AR mutations that convert anti-androgens to agonists enabling the retention of androgenic signalling in CRPC. To improve our understanding of these receptors in advanced disease we developed a physiologically-relevant model to analyse the global functionality of AR mutants in CRPC. Using the bicalutamide-activated AR_W741L/C_ mutation as proof of concept, we demonstrate that this mutant confers an androgenic-like signalling programme and growth promoting phenotype in the presence of bicalutamide. Transcriptomic profiling of AR_W741L_ highlighted key genes markedly up-regulated by the mutant receptor, including *TIPARP*, *RASD1* and *SGK1*. Importantly, *SGK1* expression was found to be highly expressed in the KUCaP xenograft model and a CRPC patient biopsy sample both of which express the bicalutamide-activated receptor mutant. Using an SGK1 inhibitor, AR_W741L_ transcriptional and growth promoting activity was reduced indicating that exploiting functional distinctions between receptor isoforms in our model may provide new and effective therapies for CRPC patients.

## INTRODUCTION

Prostate cancer (PC) is the leading cause of male cancer deaths in the western world and remains a major challenge to treat effectively [1, 2]. At presentation, PC growth is androgen-dependent hence the mainstay for treatment is hormone-ablation therapy using anti-androgens and/or androgen-deprivation therapies (ADT) [3, 4]. These act to repress the androgen receptor (AR), a member of the nuclear hormone receptor family of transcription factors that regulates expression of genes involved in prostate growth and transformation. By directly competing for androgen binding, anti-androgens such as bicalutamide, prevent activation of the AR and hence cause tumour regression [3, 5]. Unfortunately, the cancer invariably re-appears in an androgen-independent form, termed castrate-resistant PC (CRPC), that is largely fatal. Importantly, the AR signalling axis is active in this advanced stage of disease and thus remains a suitable therapeutic target [2, 6]. Indeed, the development of second generation anti-androgen and ADT therapies, such as enzalutamide [7], ARN-509 [8] and abiraterone [9] have shown promise in the treatment of CRPC. However, response rates of just 50% and the development of resistance has limited their success in the clinic [10–12].

Aberrant AR signalling is a hallmark of CRPC and is driven by numerous mechanisms including *AR* gene amplification [13], somatic receptor mutation [14, 15], expression of AR splice variants [16] and de-regulated co-factor expression [17, 18] that facilitate receptor activity in castrate conditions and contribute to treatment failure. Post-translational modification of the AR represents an additional level of receptor regulation with acetylation of key residues in the hinge region of the receptor playing a pivotal role in contact-independent growth and tumour development *in vivo* [19].

The acquisition of AR mutations during ADT, that either facilitate transcriptional activity of the receptor in the absence of androgens or switch anti-androgens to AR agonists, is a well characterised mechanism of hormone escape and has been reported to occur in upwards of 60% of CRPC patients [3, 14]. Importantly, the frequency of AR mutations in primary disease is low, but is elevated in advanced disease through therapy-specific selection of aberrantly functioning receptors [14, 15]. For example, chronic treatment with the anti-androgens bicalutamide and flutamide regularly drives selection of respective AR_W741L/C_ and AR_H874Y_/AR_T877A_ mutations that utilise the agents as agonists to promote androgenic signalling and tumour cell growth [1]. More recently, the identification of an AR_F876L_ mutation in patient samples refractory to enzalutamide and ARN-509 therapies has indicated that this is a phenomenon not limited to first-generation anti-androgens [20–22].

Modelling the function of CRPC-relevant AR mutants in their native context is challenging with most studies utilising non-PC cell lines, ectopically-expressed variant receptors and luciferase reporter-based transcriptional assays [15, 23, 24]. Outside of LNCaP cell studies, that express the AR_T877A_ mutant, there is a paucity of information on the functional dynamics and global transcriptomics of CRPC-associated AR mutants in a physiological setting that is likely to provide key biomarkers and additional treatment regimens for anti-androgen-resistant malignancies. Moreover, a major consideration for the development of next-generation AR-targeted therapies is whether they will be effective against pre-existing AR mutants in CRPC hence the development of key research tools to facilitate these studies is of high priority. To address this, we have developed a novel RNAi-rescue approach that utilises stable expression of specific AR mutants in LNCaP cells depleted of the endogenous receptor to facilitate more robust analyses of aberrant receptor signalling. Therefore, it is now possible to assess global transcriptional activity and sensitivity of CRPC-associated AR mutants to new receptor-targeting agents in a more relevant cellular context. Using the AR_W741L_ variant as a paradigm, we demonstrate that this mutant activates several endogenous AR-target genes, including *PSA* and *TMPRSS2*, and promotes a hyper-proliferative phenotype in the presence of bicalutamide; a phenomenon that can be reversed by depletion of AR_W741L_. Global transcriptomics identified a sub-set of AR_W741L_-driven genes that are markedly up-regulated compared to the endogenous receptor, including *SGK1, TIPARP* and *RASD1*. Importantly, treatment with an SGK inhibitor down-regulated bicalutamide-driven receptor activity and cell growth, suggesting this could be a novel avenue of treatment for bicalutamide-resistant patients. In all, we have successfully applied a novel AR replacement strategy to physiologically model the AR_W741L_ mutation in disease and highlighted key distinctions in receptor activity that could be therapeutically-exploited for improved CRPC treatment.

## RESULTS

### Generation of an RNAi-rescue strategy for testing AR mutant activity

**Figure 1:**
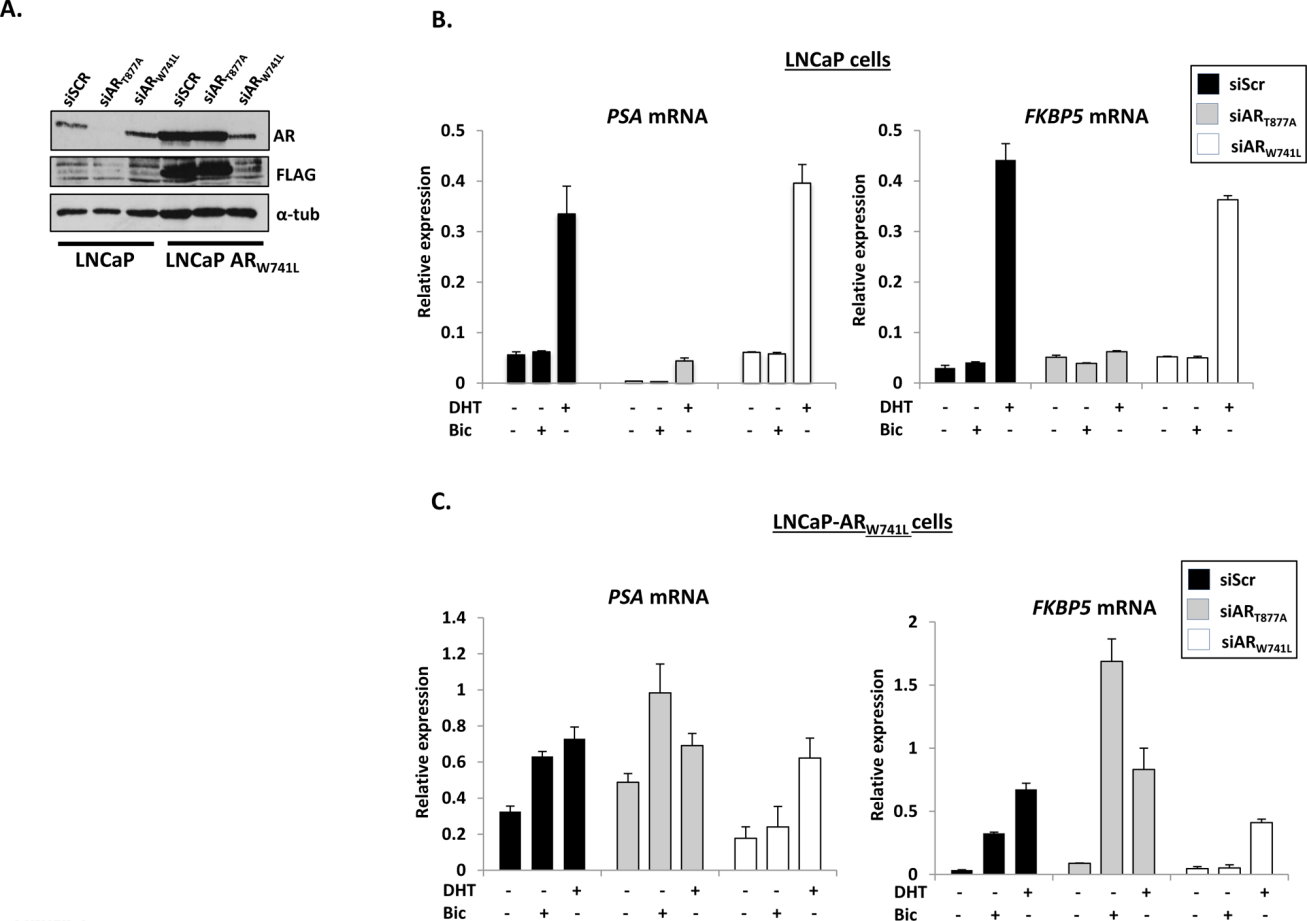
Stably-integrated AR_W741L_ in LNCaP cells up-regulates endogenous *PSA* and *FKBP5* in the presence of bicalutamide **A.** Western analysis of parental and AR_W741L_-expressing LNCaP cells depleted of either endogenous (siAR_T877A_) or ectopic (siAR_W741L_) receptors using AR, FLAG (to detect FLAG-tagged AR_W741L_) and α-tubulin antibodies. Scrambled siRNA (siScr) was used as a transfection control. Quantitative PCR analysis of *PSA* and *FKBP5* expression in parental LNCaP cells **B.** and the LNCaP-AR_W741L_ derivative **C.** depleted of either endogenous or ectopic receptors treated with 1 nM DHT or 10 nM bicalutamide for 24 hours. Data represents the mean of three independent experiments ± standard error.

There is a paucity of physiologically-relevant information on the distinct functionality of CRPC-associated AR mutants and how they drive aggressive PC malignancy. Studies to date have primarily utilised reporter-based assays incorporating ectopically-expressed mutant receptors to assess activity and sensitivity to receptor-targeting agents in AR null cell lines [14]. Although useful to demonstrate that specific CRPC-associated receptor mutants are activated by distinct ligands and down-regulated by first- and second-generation anti-androgens, as demonstrated in Supplementary Figures S1A and S1B, the failure to assess global functionality of these aberrantly functioning receptors in this context is a major problem. Improved models for examining CRPC-relevant AR mutations are therefore required. To address this, we developed a more physiological read-out for AR mutant activity using an siRNA-mediated receptor replacement strategy in the androgenic LNCaP PC cell line. Using the bicalutamide-activated AR_W741L_ as a paradigm for this study (Supplementary Figure S1), in part due to its relevance in current clinical practise, we generated an LNCaP derivative that stably expressed FLAG-tagged AR_W741L_, called LNCaP-AR_W741L_ (Figure 1A). We next designed siRNA oligonucleotides (termed siAR_T877A_) to discriminately deplete endogenous AR_T877A_ (approximately 90% knockdown) by targeting the 3′-UTR of the AR transcript that is absent in ectopically-expressed AR_W741L_ mRNA (see Supplementary Table S2 for sequences). These oligonucleotides down-regulated endogenous AR and PSA levels in LNCaP cells (Supplementary Figure S2A) and attenuated DHT-induced AR_T877A_ recruitment to *cis*-regulatory elements of the *PSA* gene (Supplementary Figure S2B). Importantly, siAR_T877A_ failed to reduce levels of ectopically-expressed FLAG-AR in PC3 cells, while an oligonucleotide targeted to the coding region of the AR (siAR) down-regulated expression of this protein (Supplementary Figure S3A and S3B). In the context of the LNCaP-AR_W741L_ cell line, as expected, siAR_T877A_ reduced endogenous AR levels, but did not affect expression of the AR_W741L_ variant (Figure 1A). Importantly, an siRNA targeted specifically to the linker region between the FLAG-tag and translation start site of the AR_W741L_ transcript markedly depleted the ectopically-expressed protein, but failed to impact on endogenous AR_T877A_.

To assess if expression of the bicalutamide-activated mutant impacts on the behaviour of the LNCaP derivative cell line, we firstly investigated the expression of known AR target genes *PSA*, *FKBP5* (Figure 1B), *KLK2* and *TMPRSS2* (Supplementary Figure S4) in the presence and absence of 1 nM DHT, 10 nM bicalutamide (pro-proliferative dose; see Supplementary Figure S5) or vehicle control and compared to parental LNCaP cells. As expected, AR-target gene expression was up-regulated by DHT, but not bicalutamide, in LNCaP cells and this effect could be negated by depletion of endogenous AR_T877A_ (Figure 1B and Supplementary Figure S4A). In contrast, both DHT and bicalutamide enhanced transcription in LNCaP-AR_W741L_ cells, and depletion of AR_T877A_ further increased bicalutamide-activated *FKBP5* and *TMPRSS2* expression (Figure 1C and Supplementary Figure S4B), suggesting a potential inhibitory role of AR_T877A_ when both receptors are co-expressed. The effect of bicalutamide on these genes was specific to the AR_W741L_ variant as knockdown using the siAR_W741L_ oligonucleotide abolished anti-androgen-driven transcription, but still enabled endogenous AR_T877A_ to drive gene expression in the presence of DHT (Figure 1C and Supplementary Figure S4B).

### Bicalutamide-activated AR_W741L_ is recruited to AR-target genes and enhances cell proliferation

**Figure 2:**
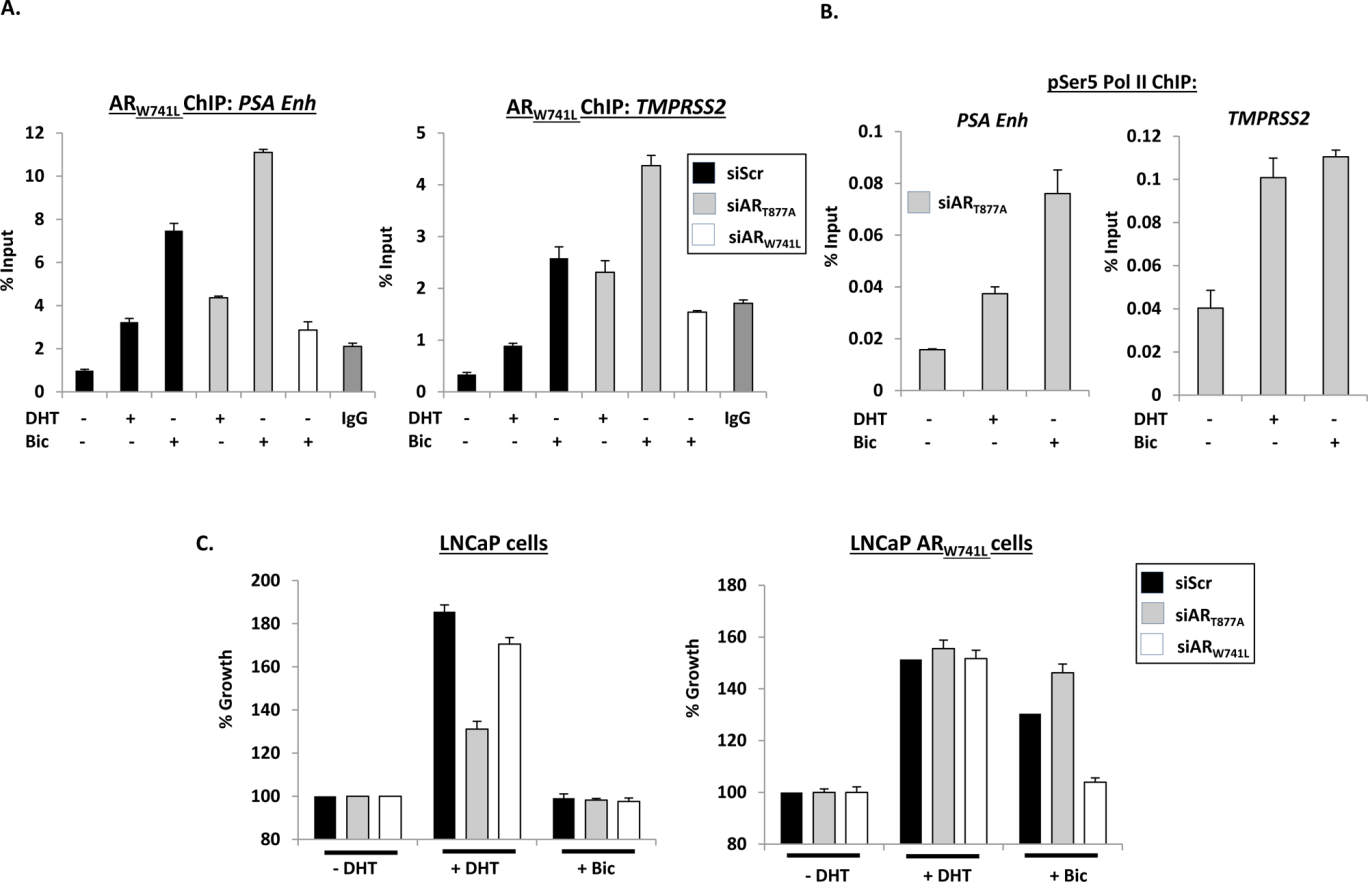
AR_W741L_ is recruited to *cis*-regulatory elements of AR-target genes and drives a pro-proliferative phenotype in response to bicalutamide **A.** LNCaP-AR_W741L_ cells depleted of either endogenous or ectopic AR were treated with 1 nM DHT or 10 nM bicalutamide for 4 hours prior to chromatin immunoprecipitation (ChIP) analysis using a FLAG antibody to immunoprecipitate FLAG-AR_W741L_. Receptor recruitment to the *PSA* enhancer (*PSA Enh*) and *TMPRSS2* promoter was assessed by quantitative PCR. **B.** LNCaP-AR_W741L_ cells depleted of endogenous receptor and treated with 1 nM DHT or 10 nM bicalutamide for 4 hours were subject to ChIP analysis using a phospho-Serine 5 RNA polymerase II antibody (pSer5 Pol II) and enrichment at *PSA* and *TMPRSS2* genes measured by quantitative PCR. **C.** LNCaP cells or the LNCaP-AR_W741L_ derivative depleted of endogenous or ectopic AR were grown in the presence of 1 nM DHT or 10 nM bicalutamide for 96 hours prior to SRB staining. Percentage growth is relative to vehicle control for each siRNA. Data is the mean of triplicate experiments ± standard error.

We next investigated recruitment of AR_W741L_ to endogenous *cis*-regulatory elements of the AR-target genes *PSA* and *TMPRSS2* in response to 1 nM DHT and 10 nM bicalutamide using a FLAG antibody. In cells expressing both AR variants (siSCR), DHT and bicalutamide activated a subtle increase in recruitment of FLAG-AR_W741L_ to these loci (Figure 2A). Consistent with gene expression data (Figure 1), depletion of endogenous AR_T877A_ markedly elevated bicalutamide-activated AR_W741L_ recruitment to the *PSA* enhancer and *TMPRSS2* promoter and this effect was diminished upon ectopic AR_W741L_ knockdown. Moreover, AR_W741L_ is also recruited to the promoter of the *KLK2* gene in response to bicalutamide and this can be attenuated with ectopic receptor knockdown (Supplementary Figure S6). Importantly, using a phospho-serine 5 RNA polymerase II antibody as a marker of transcriptional initiation, we found that DHT- and bicalutamide-activated AR_W741L_ can facilitate the assembly of the transcriptional machinery to drive expression of endogenous *PSA* and *TMPRSS2* genes (Figure 2B).

We next examined the effect of AR_W741L_ on proliferation of the LNCaP-AR_W741L_ derivative cell line in response to 1 nM DHT and 10 nM bicalutamide, and compared to parental LNCaP cells. As shown in Figure 2C, growth of LNCaP cells was increased by DHT and this effect was reduced by siAR_T877A_, but not siAR_W741L_. In contrast, growth of LNCaP-AR_W741L_ was markedly increased in the presence of both DHT and bicalutamide and only depletion of AR_W741L_ abolished bicalutamide-driven growth of these cells. To demonstrate that these findings were not an artefact of this specific clonal population of AR_W741L_-expressing cells, we tested an additional selected derivative (Clone 2) against the original (Clone 1; utilised in all previous experiments) and a control transduced cell line (LNCaP-LacZ) that does not overexpress AR_W741L_. Importantly, both Clone 1 and Clone 2 showed comparable growth stimulation in response to DHT and bicalutamide and was distinct from LNCaP-LacZ that only responded to DHT (Supplementary Figure S7).

### AR_W741L_ is inactivated by enzalutamide

**Figure 3:**
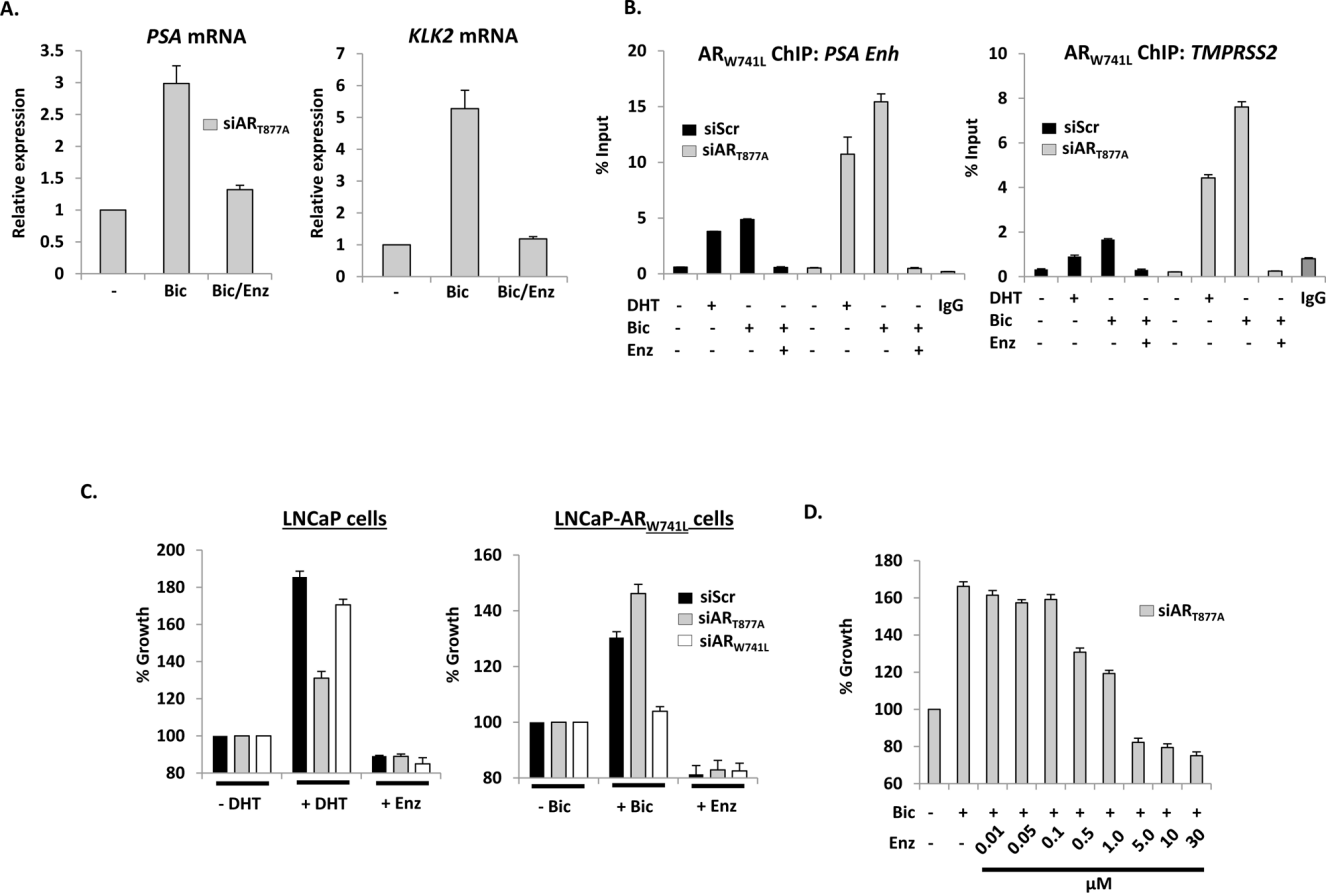
Bicalutamide-driven AR_W741L_ activity and LNCaP-AR_W741L_ cell growth is attenuated by enzalutamide **A.** LNCaP-AR_W741L_ cells depleted of endogenous AR were treated with 10 nM bicalutamide +/− 10 μM enzalutamide for 24 hours prior to quantitative analysis of AR target gene expression. **B.** LNCaP-AR_W741L_ cells transfected with siScr or siAR_T877A_ were treated for 4 hours with either 1 nM DHT or bicalutamide +/− 10 μM enzalutamide prior to ChIP and quantitative PCR analysis using primers specific to AR-target genes. **C.** LNCaP cells or the LNCaP-AR_W741L_ derivative depleted of endogenous or ectopic AR were grown in the presence of 1 nM DHT, 10 nM bicalutamide or 10 μM enzalutamide for 96 hours prior to SRB staining. Percentage growth is relative to vehicle control for each siRNA. Data is the mean of triplicate experiments ± standard error. **D.** As for (C) except LNCaP-AR_W741L_ cells depleted of endogenous AR were treated with 10 nM bicalutamide +/− increasing doses of enzalutamide to a maximum of 30 μM prior to SRB staining.

Enzalutamide has shown great promise in the clinic [25], but the fact that not all patients respond to the drug may indicate the existence of a pre-determinant, such as an AR mutant, that compromises enzalutamide efficacy [20, 21]. Given that enzalutamide in many cases is given as a second-line therapy post-bicalutamide treatment, it is therefore important to establish if the AR_W741L_ mutant is sensitive to enzalutamide in our more robust CRPC model system. To this end, we firstly assessed the effect of 10 μM enzalutamide on bicalutamide-activated AR_W741L_ transcriptional activity in LNCaP-AR_W741L_ cells. As expected, bicalutamide up-regulated *PSA* and *KLK2* gene expression, and importantly, this was reduced to basal levels upon administration of enzalutamide (Figure 3A). Furthermore, ChIP analysis using a FLAG antibody demonstrated that enzalutamide markedly diminished bicalutamide-activated AR_W741L_ recruitment to *cis*-regulatory elements of the *PSA*, *TMPRSS2* and *KLK2* genes (Figure 3B and Supplementary Figure S8).

In proliferation assays, we showed that enzalutamide reduced growth of both parental and AR_W741L_-expressing LNCaP cells (Figure 3C and Supplementary Figure S9). Importantly, enzalutamide attenuated bicalutamide-driven proliferation of LNCaP-AR_W741L_ cells (Figure 3D) indicating that this second-generation anti-androgen is likely to be effective in CRPC patients harbouring the AR_W741L_ mutation.

### AR_W741L_ drives an androgenic signalling programme similar to AR_T877A_

**Figure 4:**
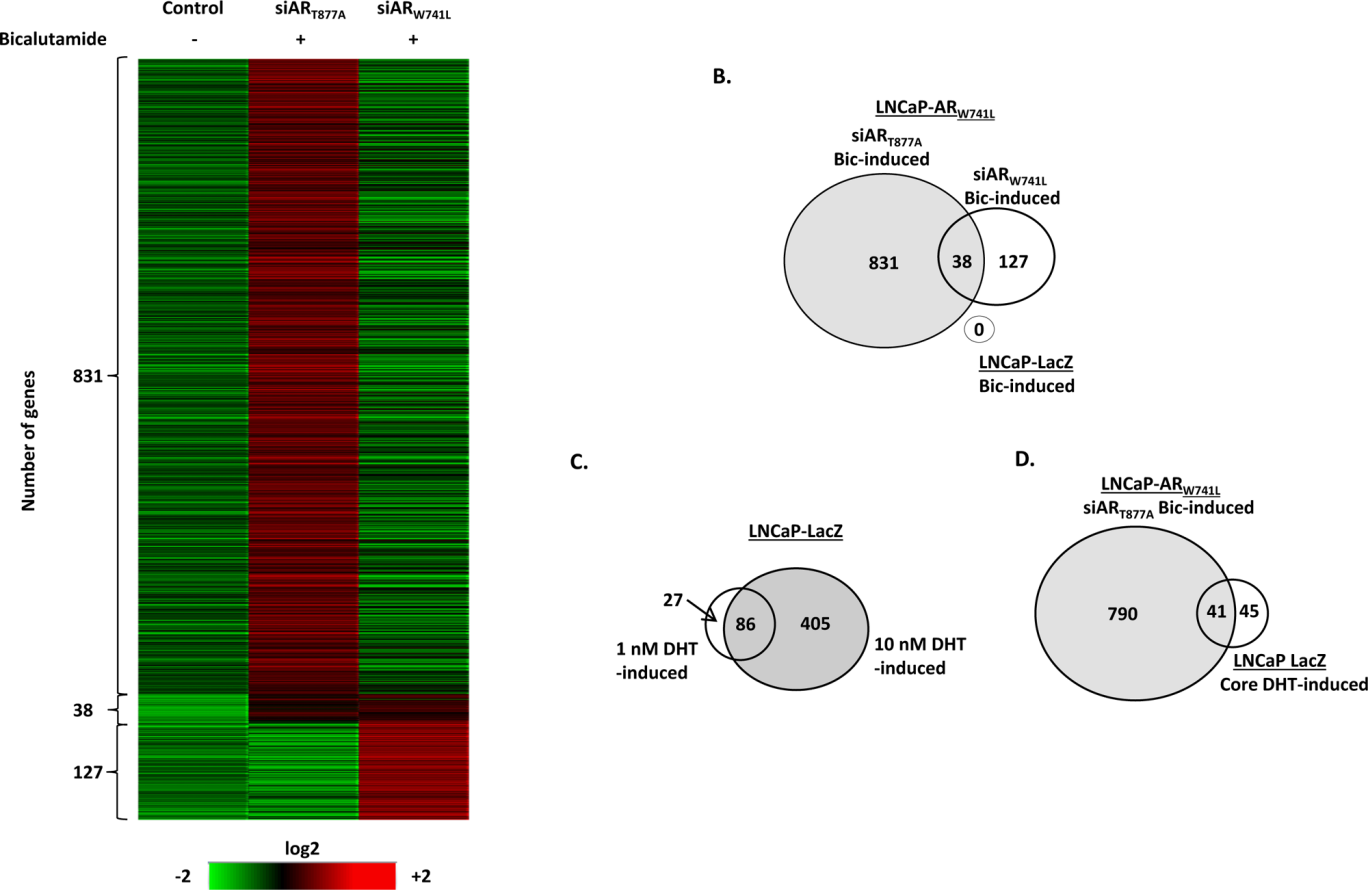
Bicalutamide activates an androgenic gene signature programme in LNCaP-AR_W741L_ LNCaP-AR_W741L_ cells depleted of either endogenous or ectopic AR were treated with 10 nM bicalutamide for 24 hours prior to micro-array analysis. Resultant transcriptome was compared to control LNCaP-LacZ cells treated with 10 nM bicalutamide. Genes with a fold increase of >1.5 were considered to be ligand regulated and included in the analysis. Genes with a bicalutamide-induced fold increase of >2.0 in the AR_W741L_-depleted experimental arm were eliminated from the core list of bicalutamide-induced genes. Data is presented as a heat map **A.** and Venn diagram **B, C.** LNCaP-LacZ control cells were treated with either 1 or 10 nM DHT for 24 hours prior to micro-array analysis to identify a core of DHT-regulated genes between each condition. **D.** Overlap between bicalutamide-induced AR_W741L_ transcriptome and the identified set of 86 core DHT-up-regulated genes in the LNCaP-LacZ cells.

We next tested the utility of this model to provide much needed information on the global transcriptional targets of the CRPC-relevant AR_W741L_ mutant. Using LNCaP-AR_W741L_ cells depleted of endogenous AR_T877A_, the expression profile of AR_W741L_ in response to bicalutamide was compared to vehicle treated controls and a total of 869 genes were identified as being upregulated >1.5 fold (Figure 4A and 4B; siAR_T877A_). This threshold was chosen as it fell within fold change cut-offs that have been used in previous publications investigating AR signalling profiles [26–28]. As a control, LNCaP-LacZ cells were treated with 10 nM bicalutamide and the resultant transcriptome was compared to the gene list identified in LNCaP-AR_W741L_ cells. Importantly, no genes were identified in the LNCaP-LacZ control line that exhibited >1.5 fold up-regulation in the presence of bicalutamide, suggesting that the identified set of bicalutamide-induced genes in the LNCaP-AR_W741L_ cells were specific to the ectopically-expressed receptor (Figure 4A and 4B). To refine the list of core bicalutamide-induced genes further, we incorporated an additional control in which the expression profile of bicalutamide-treated LNCaP-AR_W741L_ cells depleted of AR_W741L_ was examined relative to siAR_T877A_ vehicle and bicalutamide-stimulated experimental arms (Figure 4A and 4B; siAR_W741L_). Genes that exhibited > 2 fold increase in expression following bicalutamide treatment in AR_W741L_-depleted cells were deemed to be bicalutamide-independent. We identified 38 genes matching to the 869 core data set that were subsequently eliminated (Figure 4A and 4B); resulting in a refined gene set of 831 genes whose expression were considered to be specifically driven by bicalutamide-activated AR_W741L_. The full list of bicalutamide-induced genes are listed in Supplementary Table S4 and include characterised AR-target genes such as *PSA, KLK2, TMPRSS2, NKX3.1, KCNN2* and *SPOCK1*

We next compared the bicalutamide-driven transcriptome of LNCaP-AR_W741L_ with the DHT-stimulated LNCaP-LacZ cell line derivative. In response to 1 and 10 nM DHT, microarray analysis revealed 113 and 491 up-regulated genes, respectively, relative to vehicle control (Figure 4C; Supplementary Tables S5 and S6). Comparison of both gene lists found 86 common genes (76% and 17% of 1 nM and 10 nM DHT up-regulated genes, respectively), highlighting a core set of androgen-regulated target genes that are activated in response to both 1 nM and 10 nM DHT (Figure 4C). As expected, a greater number of genes were activated in response to 10 nM DHT than 1 nM DHT, including *FKBP5* (Supplementary Figure S10).

Direct comparison of the bicalutamide-induced LNCaP-AR_W741L_ expression profile to the core set of androgen-regulated genes in LNCaP-LacZ, found that 41 of the 86 genes (48%) were common to both lists (Figure 4D). For example, *PSA* (*KLK3*) and *KLK2*, which were found to be the most DHT-stimulated in LNCaP-LacZ cells were also elevated in response to bicalutamide in LNCaP-AR_W741L_ cells and is consistent with LNCaP-AR_W741L_ gene expression data (Figure 1C). We next compared the bicalutamide-activated AR_W741L_ transcriptome with two published androgenic gene signatures acquired from LNCaP cells [27, 29]. Of the respective 21 and 79 androgen-induced genes identified in the two studies, 15/21 (71%) and 52/79 (66%) matched directly to genes identified in the bicalutamide-activated LNCaP-AR_W741L_ data-set (Supplementary Figure S11; see Supplementary Table S7), indicating robust commonality between the bicalutamide-induced AR_W741L_ and DHT-stimulated AR_T877A_ transcriptional programmes.

### Exploiting AR_W741L_-driven *SGK1* expression to inactivate CRPC cell growth

**Figure 5:**
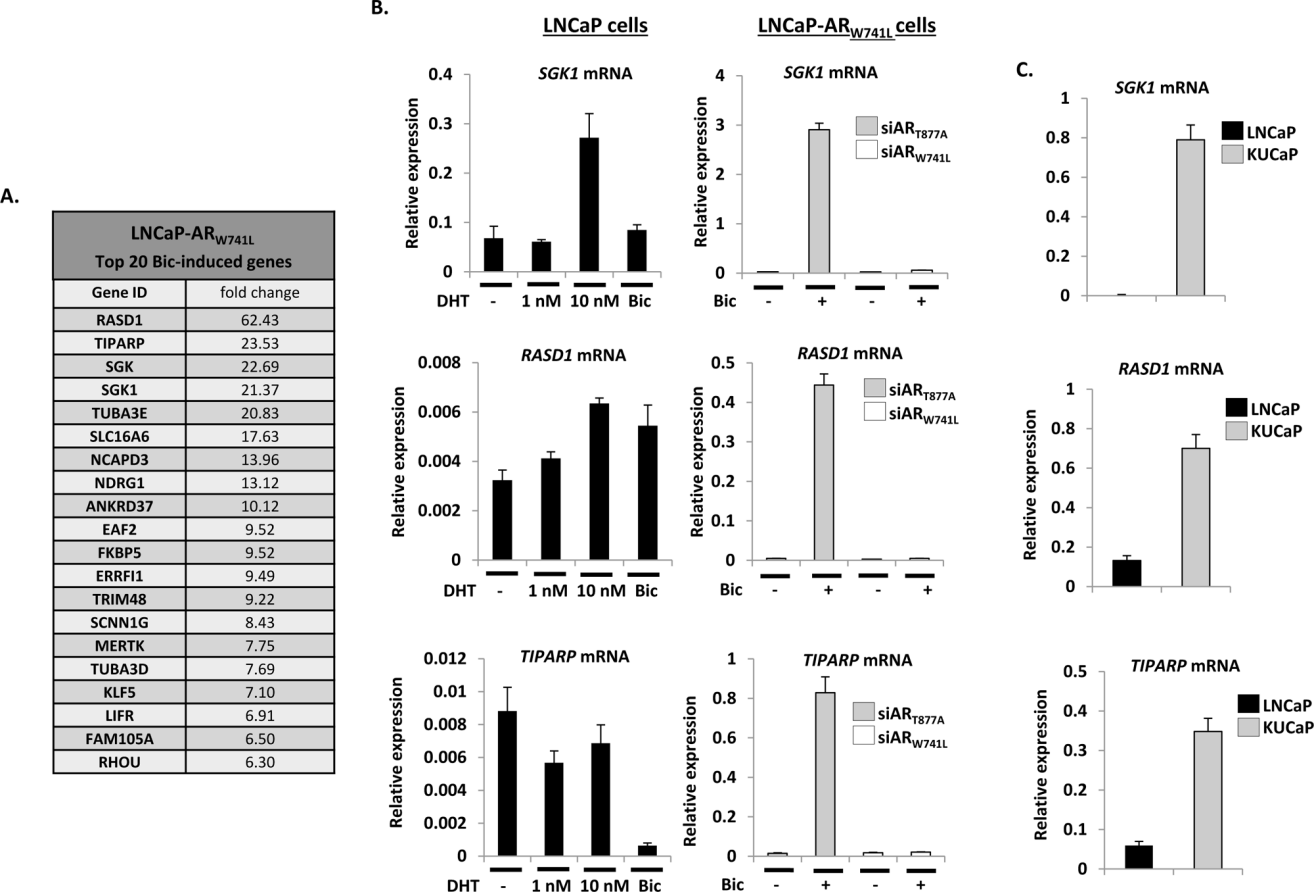
AR_W741L_ markedly up-regulates genes distinct from endogenous AR_T877A_ **A.** Top 20 bicalutamide-induced genes identified in siAR_T877A_-transfected LNCaP-AR_W741L_ cells. **B.** LNCaP cells and LNCaP-AR_W741L_ depleted of endogenous or ectopic receptors, were treated with either DHT (1 and 10 nM) or 10 nM bicalutamide for 24 hours prior to quantitative analysis of *SGK1*, *RASD1* and *TIPARP* expression. **C.** Expression analysis of the same genes was compared between LNCaP cells and the AR_W741C_-expressing KUCaP xenograft model.

We next focused our attention on identifying AR_W741L_-driven genes that were significantly elevated in response to bicalutamide and distinct from our DHT-stimulated LNCaP-LacZ transcriptome to define biomarkers of this specific CRPC-associated AR mutation and potentially highlight avenues for therapeutic exploitation. Of the top 20 bicalutamide up-regulated genes in LNCaP-AR_W741L_ cells (Figure 5A), several were well known AR target genes, including *FKBP5* and *NDRG1*, and are elevated in response to 10 nM DHT treatment in our LNCaP-LacZ control line (Supplementary Table S6). Importantly, array data indicated that *TIPARP* (*TCDD-inducible poly(ADP-ribose) polymerase*), *RASD1* (*Ras dexamethasone-induced 1*) and *SGK1* (*serum- and glucocorticoid-regulated kinase 1*) were exclusively and markedly up-regulated by bicalutamide in the LNCaP-AR_W741L_ cell line compared to the DHT-stimulated control LNCaP-LacZ derivative (data not shown). Robust bicalutamide-mediated up-regulation of *TIPARP* (57-fold), *RASD1* (93-fold) and *SGK1* (109.5-fold) was validated by QPCR, and demonstrated to be exclusively mediated by AR_W741L_ as depletion of this mutant by siAR_W741L_ completely abrogated gene expression (Figure 5B). In contrast, treatment of LNCaP-LacZ cells with a dose-range of DHT (Figure 5B and Supplementary Figure S12) only modestly increased *SGK1* (5-fold) and *RASD1* (2-fold) expression and failed to elevate *TIPARP* transcript levels while, as expected, *PSA* was greatly up-regulated (Supplementary Figure S13). Moreover, analysis of an additional LNCaP derivative that ectopically expresses wild-type AR (LNCaP-wtAR) to levels comparable to that of AR_W741L_ (data not shown), demonstrated modest enhancement of *SGK1*, *TIPARP* and *RASD1* transcription in response to DHT stimulation with respective 4.5-, 8.6- and 5.9-fold induction, indicating that the robust up-regulation of the three genes in LNCaP-AR_W741L_ cells is not due to the phenomenon of elevated cellular AR levels (Supplementary Figure S14).

To investigate further the discriminate enhancement of *TIPARP*, *RASD1* and *SGK1* by AR_W741L_, we profiled expression of these genes in the KUCaP xenograft CRPC model. This xenograft was derived from a liver metastasis present in a bicalutamide-resistant CRPC patient and exclusively expresses the AR_W741C_ mutation [30]. As shown in Figure 5C, expression of *TIPARP*, *RASD1* and *SGK1* were markedly elevated in KUCaP cells compared to the LNCaP line confirming that key distinctions exist between transcriptomes of AR_W741L_ and AR_T877A_ that could be important in the pathobiology of disease in CRPC patients harbouring the bicalutamide-resistant mutation.

**Figure 6:**
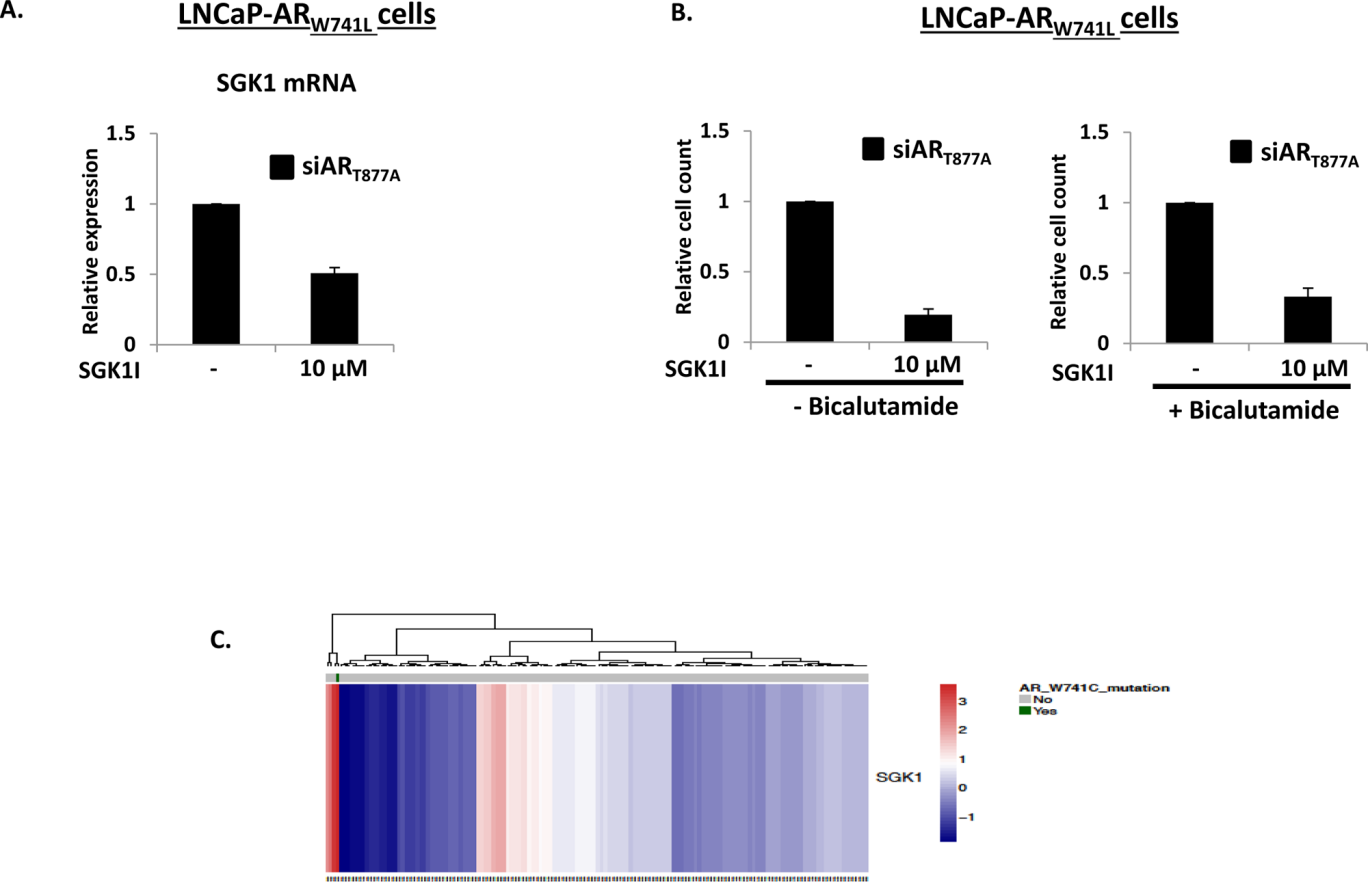
SGK1 inhibition reduces AR_W741L_ activity and attenuates LNCaP-AR_W741L_ proliferation **A.** LNCaP-AR_W741L_ cells depleted of AR_T877A_ were treated with 10 μM SGK inhibitor GSK650395 for 24 hours prior to quantitative analysis of *SGK1* expression. **B.** As above, but cells were treated with GSK650395 for 96 hours in the presence and absence of 10 nM bicalutamide prior to proliferation analysis. **C.** *In silico SGK1, TIPARP and RASD1* expression profiling in 150 primary prostate cancer specimens (28); AR_W741C_-expressing patient sample is indicated in green.

To address if the distinct gene-set of AR_W741L_ could be exploited to provide key targets for CRPC therapy, we focussed on the potent up-regulation of *SGK1* expression by this mutant receptor. Given that a previous study indicated that inactivation of SGK1 using the selective inhibitor GSK650394 reduces *SGK1* expression and LNCaP cell growth [31], we hypothesised that the LNCaP-AR_W741L_ derivative may be sensitive to this agent as they demonstrate markedly elevated *SGK1* expression compared to our LNCaP control cells (Figure 5B). To this end, we assessed AR_W741L_-driven expression of *SGK1* in the presence and absence of 10 μM GSK650395; a dose demonstrated to down-regulate SGK1 transcript levels in LNCaP cells [31]. As shown in Figure 6A, treatment of LNCaP-AR_W741L_ cells with GSK650395 reduced expression of *SGK1* by approximately 50% indicating that activity of the bicalutamide-activated receptor is potentiated by SGK1 and attenuation of the associated kinase activity down-regulates AR_W741L_-mediated transcription. Importantly, SGK1 inhibition reduced LNCaP-AR_W741L_ proliferation by approximately 70% (Figure 6B) indicating SGK1 is a key down-stream effector of AR_W741L_-driven cell growth.

Finally, we conducted a systematic *in silico* analysis of a comprehensive PC cohort containing 150 primary tumour samples [32] and identified one patient biopsy expressing the AR_W741C_ mutation. Consistent with our findings from the LNCaP-AR_W741L_ derivative and KUCaP xenograft, *SGK1* gene expression was found to be significantly up-regulated in this sample compared to all other samples suggesting that this may be a *bona fide* biomarker for aberrant AR_W741L_ function (Figure 6C and Supplementary Figure S15) and manipulating activity of this enzyme may provide an additional means of treatment for CRPC patients resistant to bicalutamide.

## DISCUSSION

The selection of AR mutations during androgen-depravation therapy is a well-defined mechanism of therapy resistance that, to date, has been reported to occur in upwards of 60% advanced CRPC patients [14, 15]. Although unclear, this figure may increase further due to two key developments: (i) the utility of more sensitive approaches for detecting mutations in both diagnostic and basic research [32], and (ii) ease of access to disseminated disease through the study of circulating tumour cells that offers a non-invasive means for *AR* sequencing in CRPC [33, 34]. Importantly, the presence of mutant AR in CRPC poses particular clinical challenges as many of the identified mutations promote promiscuous receptor activity that enable androgenic signalling by non-conventional ligands, including bicalutamide, flutamide [14], and more recently, enzalutamide/ARN-509 [10, 21]; hence limiting available treatment options in advanced disease. From a biological perspective, our understanding of the global functioning of these aberrant receptors is remarkably limited. Outside of studies in the PC cell lines LNCaP, that harbour the AR_T877A_ mutation, and CWR22RV1, that express AR_H874Y_ and also numerous alternatively spliced AR isoforms, there is a reliance upon transient expression of mutant receptors in non-androgenic cell lines to study, in most cases, the transcriptional dynamics of these proteins [23, 24]. Although useful, these experiments offer little or no insight into global transcriptomics of AR mutants and lack physiological context. Given the prevalence of AR mutations in advanced CRPC, defining their activity in more robust and disease-relevant models is imperative to help improve our understanding of these receptors and to potentially exploit their distinct activities for the development of new PC treatments.

To this end, we developed a novel RNAi-rescue system to enable the study of AR mutants in the physiological background of LNCaP cells that have been depleted of the endogenous receptor. Using the bicalutamide-activated AR_W741L_ as a proof of concept mutation, that is also clinically-relevant, we generated an LNCaP cell line derivative that stably-expressed this mutant receptor (LNCaP-AR_W741L_) and developed key siRNAs to deplete either endogenous or ectopic AR_T877A_ and AR_W741L_, respectively. Using this model, we demonstrated that in the presence of bicalutamide, AR_W741L_ associates with *cis*-regulatory elements of several AR target genes, including *PSA* and *TMPRSS2*, and facilitates their expression. Significantly, these effects were attenuated upon depletion of the ectopic receptor indicating that the bicalutamide-driven functionality of AR_W741L_ previously characterised in AR null cell line studies (Supplementary Figure S1 and [35]) has been phenocopied in LNCaP cells with promotion of chromatin-binding and endogenous target gene expression by the anti-androgen akin to the DHT-activated endogenous AR_T877A_ isoform. Interestingly, data from both chromatin immunoprecipitation and candidate gene expression analysis (*PSA*, *TMPRSS2*, *KLK2*) experiments demonstrated an inhibitory effect of AR_T877A_ on bicalutamide-activated AR_W741L_ when both receptor isoforms were expressed (siScr control; Figures 1 and 2). We hypothesise that dimerization between the two distinct proteins may occur in the presence of bicalutamide and impact on their activity; homodimers of AR_W741L_ will be transcriptionally potent, while AR_T877A_-AR_W741L_ heterodimers are likely to be functionally compromised. By depleting endogenous AR_T877A_ from LNCaP cells, the equilibrium is pushed toward the generation of active AR_W741L_ dimers to promote more robust AR-target gene binding and transcription.

To further support the utility of this RNAi-rescue system to model distinct CRPC-relevant AR mutations, we demonstrated that the AR_W741L_ mutant promoted growth of the LNCaP derivative line in the presence of bicalutamide. The pro-proliferative effect of bicalutamide was solely driven by the activity of AR_W741L_ confirming that the ectopic receptor replaces the activity of AR_T877A_ and illustrates the ability for this model to recapitulate conditions of a bicalutamide-resistant CRPC disease state.

From these promising indications, we next tested the impact of the second-generation anti-androgen enzalutamide on mutant activity and growth of the LNCaP-AR_W741L_ derivative. This is a particularly important experiment when one considers that new AR-targeting compounds will be applied to advanced, ADT-resistant CRPC that are likely to harbour pre-existing AR mutations [3]; hence defining efficacy of agents toward CRPC-relevant receptor mutations in a physiological model system is critical for optimal pre-clinical drug testing. In both the presence and absence of bicalutamide, AR_W741L_-chromatin binding, endogenous target gene expression and LNCaP-AR_W741L_ growth was down-regulated by enzalutamide indicating that this mutation is sensitive to the second-generation anti-androgen. From this study, it is therefore possible to predict that patients refractory to bicalutamide through the selection of specific AR mutations are likely to demonstrate a clinical response to enzalutamide. However, given that up to 50% of patients do not respond to enzalutamide [7, 25], it is likely that other pre-determinants in advanced disease may compromise AR-targeting agent efficacy; the selection of other distinct mutations during first-line ADT could represent one such mechanism of new treatment failure. Importantly, our novel rescue system would enable the robust modelling of other CRPC-relevant receptor mutants that could facilitate patient stratification towards those predicted to receive benefit from sequential anti-AR targeted therapy and those that would not.

In keeping with the utility of this model as an important pre-clinical tool to identify distinctions in the functionality of disease-associated AR mutations, we hypothesised that global transcriptomic profiling of AR_W741L_ would provide key gene targets that could be exploited for novel CRPC treatment strategies. Examination of the bicalutamide-activated AR_W741L_ target gene-set against a control transduced LNCaP derivative (LNCaP-LacZ) identified a series of bicalutamide-induced genes; many of which were known AR-target genes including *PSA*, *KLK2* and *TMPRSS2.* Furthermore, although the number of bicalutamide-activated genes were in excess of those controlled by DHT in our control cells and those reported in Hieronymous *et al*., [29] and Nelson *et al*., [27] there was considerable overlap in the bicalutamide- and androgen-activated transcriptomes supporting the concept that AR_W741L_ maintains a common androgenic expression programme. Importantly, a number of robustly up-regulated bicalutamide-dependent genes were identified by micro-array in LNCaP-AR_W741L_ cells that were not greatly enhanced by DHT in the LNCaP-LacZ line, including *TIPARP*, *RASD1* and *SGK1*. Depletion of ectopic receptor in LNCaP-AR_W741L_ cells attenuated this bicalutamide-driven response indicating that these genes are potentially discriminate targets of AR_W741L_. This dramatic and selective activity of the AR_W741L_ mutant is intriguing and may be a consequence of subtle allosteric re-organisation of the receptor that permits hyper-activation of the receptor at distinct genomic loci. By repositioning the C-terminal activation function-2 (AF-2) domain of the receptor, the leucine residue may enable more robust interaction with selective co-regulators to drive acetylation and methylation of the AR to enhance inherent transcriptional activity [19, 36].

In contrast to our data, however, *SGK1* has been identified to be robustly up-regulated by endogenous AR_T877A_ in LNCaP cells and the gene product found to positively reinforce androgenic signalling [31, 37]. The discrepancy between our data and these findings is intriguing and may reflect key differences in experimental design and performance, as although we did detect a 3-fold and 4.5-fold enhancement of *SGK1* expression in response to 10 nM DHT by quantitative PCR in the respective LNCaP-LacZ and LNCaP-wtAR cell derivatives, it was significantly less than previous reported.

Importantly, the same study provided evidence that inactivation of SGK1 with the selective small molecule inhibitor GSK650395 was able to attenuate LNCaP cell proliferation in the presence of DHT [31]. Therefore, given that LNCaP-AR_W741L_ cells demonstrated marked up-regulation of *SGK1* expression in response to bicalutamide, we speculated that they may also be sensitive to SGK1 inhibition. Consistent with the reported phenomenon in parental LNCaP cells [31], we found that AR_W741L_-driven *SGK1* expression was down-regulated in response to GSK650395 treatment, suggesting that ectopic receptor function is dependent upon SGK1-mediated kinase activity. Furthermore, proliferation of both LNCaP and LNCaP-AR_W741L_ cells was significantly down-regulated upon SGK1 inhibition; with similar IC_50_ values for both cell lines in response to GSK650395 (Supplementary Figure S16). Importantly, in the LNCaP-AR_W741L_ cells, this anti-proliferative effect was maintained in the presence of a pro-proliferative dose of bicalutamide. We have therefore identified a strongly up-regulated AR_W741L_ target gene that may offer an avenue for therapeutic exploitation in bicalutamide-refractory CRPC. Although these observations are based on our rescue model system, evidence from the patient-derived KUCaP xenograft and a single patient biopsy sample, that both harbour the bicalutamide-activated AR_W741C_ mutation [30, 32], is in agreement with our findings of elevated *SGK1* expression and suggests utility of our RNAi-rescue approach to define global functionality of the AR_W741L_ mutation.

In all, we have established a novel LNCaP cell line-based strategy to model CRPC-relevant mutations to provide important information on receptor dynamics and global gene signalling in response to distinct activating ligands.

## MATERIALS AND METHODS

### Luciferase reporter, quantitative PCR and western analyses

Luciferase assays were performed in HEK293T cells grown in steroid-depleted media as described in [38] utilising the p(ARE)_3_ reporter and pFLAG-AR, -AR_W741L_ and –AR_H874Y_. Quantitative PCR was used to assess expression of endogenous AR-and AR_W741L_-target genes (see Supplementary Table S1 for primer sequences) using cDNA generated from Trizol-mediated RNA extractions as described in [39]. Western blotting was performed as described in [40] using antibodies listed in Supplementary Table S3.

### Cell proliferation assays

Sulforhodamine B (SRB) assays were performed according to [41]. Briefly, 5 × 10^3^ LNCaP or LNCaP-AR_W741L_ cells per well of a 96-well plate grown in steroid-depleted conditions were transfected with siScr, siAR_T877A_ or siAR_W741L_ for 96 hours as described above before fixing in trichloroacetic acid for 1 hour at 4°C. Cells were washed and subsequently stained with 0.4% SRB dissolved in 1% acetic acid. Plates were air dried at room temperature, after which bound SRB was dissolved in 10 mM Tris-HCl, pH 10.8. Absorbance was measured at 570 nm using a 96-well plate reader (BioRad). For drug treatments, 10 nM bicalutamide, 1 nM DHT, or increasing doses of enzalutamide, were administered for 96 hours prior to SRB assay.

For additional Methods please refer to accompanying Supplementary Information.

## SUPPLEMENTARY MATERIALS AND METHODS


