# Supplementary material for: Development and exploitation of a novel mutant androgen receptor modelling strategy to identify new targets for advanced prostate cancer therapy
Source: Oncotarget. 2015 Jul 20;6(28):26029–40. doi: 10.18632/oncotarget.4347 (PMC4694883; doi:10.18632/oncotarget.4347)
Supplement: Supplementary file 1 [file oncotarget-06-26029-s001.pdf]

SUPPLEMENTARY MATERIALS AND METHODS

Plasmids, lentivirus and LNCaP-AR$_{W741L}$ production

The p(ARE)$_3$-luciferase reporter has been previously described (34) and pFLAG-wtAR was a kind gift from Ralf Janknecht (Oklahoma University, Rochester, MN). pFLAG-AR$_{T877A}$ and -AR$_{W741L}$ were generated in house by site-directed mutagenesis (Aglient Technologies) using pFLAG-wtAR as a template (see Supplementary Table S1 for primer sequences). FLAG-AR$_{W741L}$ and FLAG-wtAR were subsequently cloned into the pENTR™/D-TOPO vector (Life Technologies) before sub-cloning into the pLenti6.3/V5-DEST (Life Technologies) to generate the pLenti-FLAG-AR$_{W741L}$ and pLenti-FLAG-wtAR, respectively.

The ViraPower Lentiviral Expression System kit (Life Technologies) was used to generate viral particles as per the manufacturer's guidelines. Briefly, 5×10^6 HEK293T cells grown in reduced serum Opti-MEM I medium (Life Technologies) were transfected with either pLenti-FLAG-AR$_{W741L}$ or pLenti-FLAG-wtAR and the ViraPower packaging mix using Lipofectamine 2000 (Life Technologies). After 24 hours, culture media was replaced with fresh full media and incubated for an additional 72 hours, prior to virus collection and concentration using ultracentrifugation at 26,500 g for 2 hours at 4°C. The viral pellet was then resuspended in full media and stored at –80°C.

Using a multiplicity of infection (MOI) of 0.3 and 0.1, LNCaP cells were transduced with either pLenti-AR$_{W741L}$, pLenti-wtAR or empty pLenti-LacZ control virus and several stably expressing clones of each, termed LNCaP-AR$_{W741L}$, LNCaP-wtAR and LNCaP-LacZ, respectively, were selected with 10 µg/ml blasticidin, expanded and, for LNCaP-AR$_{W741L}$ and LNCaP-wtAR derivatives, ectopic AR expression was determined by western blotting using a FLAG antibody. Depletion of endogenous AR was initially optimised with three 3′-UTR-targeting siRNAs (labelled siAR$_{T877A}$-1/2/3; see Supplementary Table S2 for all siRNA sequences) and all subsequent experiments utilised the siAR$_{T877A}$-2 oligonucleotide (referred to as siAR$_{T877A}$ in the manuscript). To knockdown ectopically-expressed AR$_{W741L}$, an oligonucleotide targeting the linker sequence between the FLAG-tag and receptor cDNA was utilised (labelled siAR$_{W741L}$). All siRNA transfection experiments were conducted as described in (35) using RNAiMax (Life Sciences) with the exception that we utilised a double knockdown strategy to maintain and maximise knockdown by approximately 90% for up to 5 days. All experiments incorporated a control scrambled sequence siRNA (siScr).

LNCaP, PC3 and HEK293T cells were purchased from American Type Culture Collection (ATCC). cDNA from the KUCaP xenograft model was a kind gift from Dr Osamu Ogawa.

Chromatin immunoprecipitation (ChIP) analysis

ChIP assays were performed as described in (35) utilising antibodies listed in Supplementary Table 3. Quantitative PCR of resultant ChIP'd DNA was performed using primers to several AR-target genes (see Supplementary Table 2 for sequences). For ChIP experiments investigating recruitment of AR$_{W741L}$ to target genes, LNCaP-AR$_{W741L}$ cells grown in steroid-depleted media were transfected with either siAR$_{T877A}$, siAR$_{W741L}$ and siScr control oligonucleotides for 68 hours prior to 4 hour stimulation with either 1 nM dihydrotestosterone (DHT), 10 nM bicalutamide, 10 µM enzalutamide or vehicle control prior to chromatin preparation.

Micro-array analysis

RNA was extracted from both LNCaP-AR$_{W741L}$ cells subjected to endogenous or ectopic AR knockdown, and LNCaP-LacZ cells grown in steroid-depleted media treated with vehicle, 1 nM DHT, 10 nM bicalutamide as described above. Triplicate samples were hybridised onto an Illumina HT12 v4 BeadChip Array (performed by The Wellcome Trust Centre for Human Genetics, Oxford University) and analysed using GenomeStudio (Illumina) to assess differential gene expression incorporating quantile normalisation and multiple sample testing correction using Benjamini and Hochberg false discovery rate ($p < 0.05$). Genes were filtered further using custom equations to exclude genes with an insignificant detection p-value as recommended by Illumina ($p < 0.05$). Compound treated samples were expressed as fold-change by dividing the average sample signal by vehicle treated controls. Data is represented as the mean of three independent experiments. Genes without a gene symbol/ID and those with locus specific entries were removed from the overall analysis. Additionally, as ligand-induced genes were the primary end-point, only genes that exhibited increased expression were analysed further.

For *SGK1* expression analysis from the Taylor *et al.*, patient biopsy data-set (28), the normalised log2 mRNA expression data was downloaded from the MSKCC Prostate Cancer Genomics Data Portal: http://cbio.mskcc.org/prostate-portal/. Samples representing normal tissue and cell lines/xenografts were removed from the dataset. This leaves 150 prostate cancer samples, with only 1 expressing the AR$_{W741C}$ mutation (PCA0188). The resampling approach was used to estimate the probability of obtaining expression levels of *SGK1* as high as those observed in the AR$_{W741C}$ mutant sample from the set of 149 non-AR$_{W741C}$ mutant

cancer samples. We resampled one biopsy data-set at a time from the non-mutant samples (with replacement) one million times at random. In each iteration, the expression value of that gene from the non-AR_{W741C} mutant biopsy was compared to the expression value of the same gene from the AR_{W741C} mutant sample. After one million resamplings, we calculated the probability (*p*-value) of non-AR mutant biopsies expressing a specific gene greater than in the AR_{W741C} biopsy. This is the number of resamplings that have a greater level of expression than those of the expression of the same respective genes in the mutant sample divided by 1,000,000.

SUPPLEMENTARY FIGURES AND TABLES

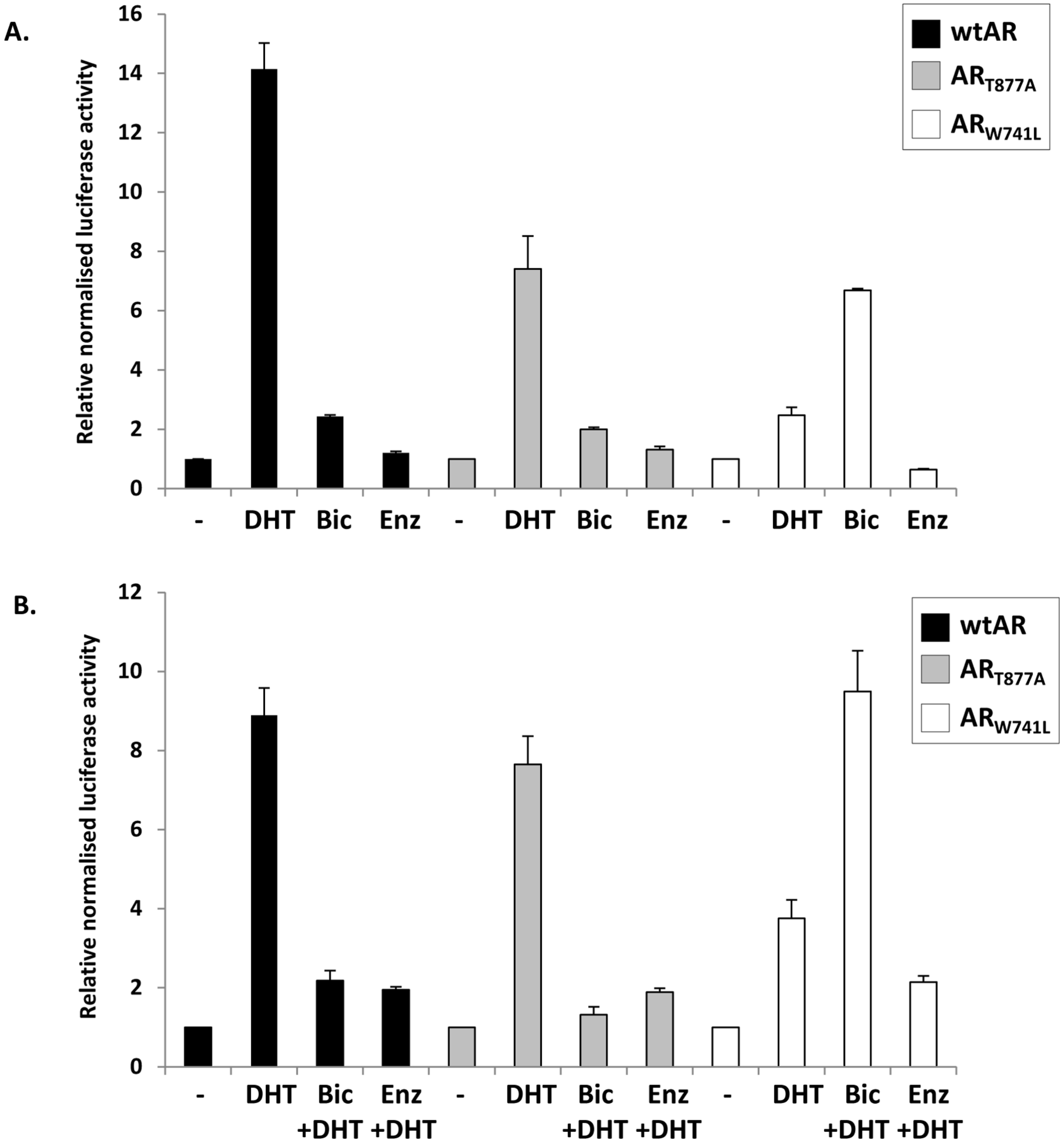

Supplementary Figure S1: AR_{W741L} demonstrates distinct activation profiles to wild-type AR and AR_{T877A}. A. PC3 cells transfected with wild-type AR (wtAR), AR_{W741L} and AR_{T877A} and a receptor-responsive luciferase reporter were treated with single agent doses of 10 nM DHT, 10 µM bicalutamide and 10 µM enzalutamide for 24 hours before luciferase expression analysis. Data represents three independent repeats ± standard error. **B.** As for (A), but cells were cultured for 24 hours in dual DHT +/− bicalutamide or enzalutamide treatments.

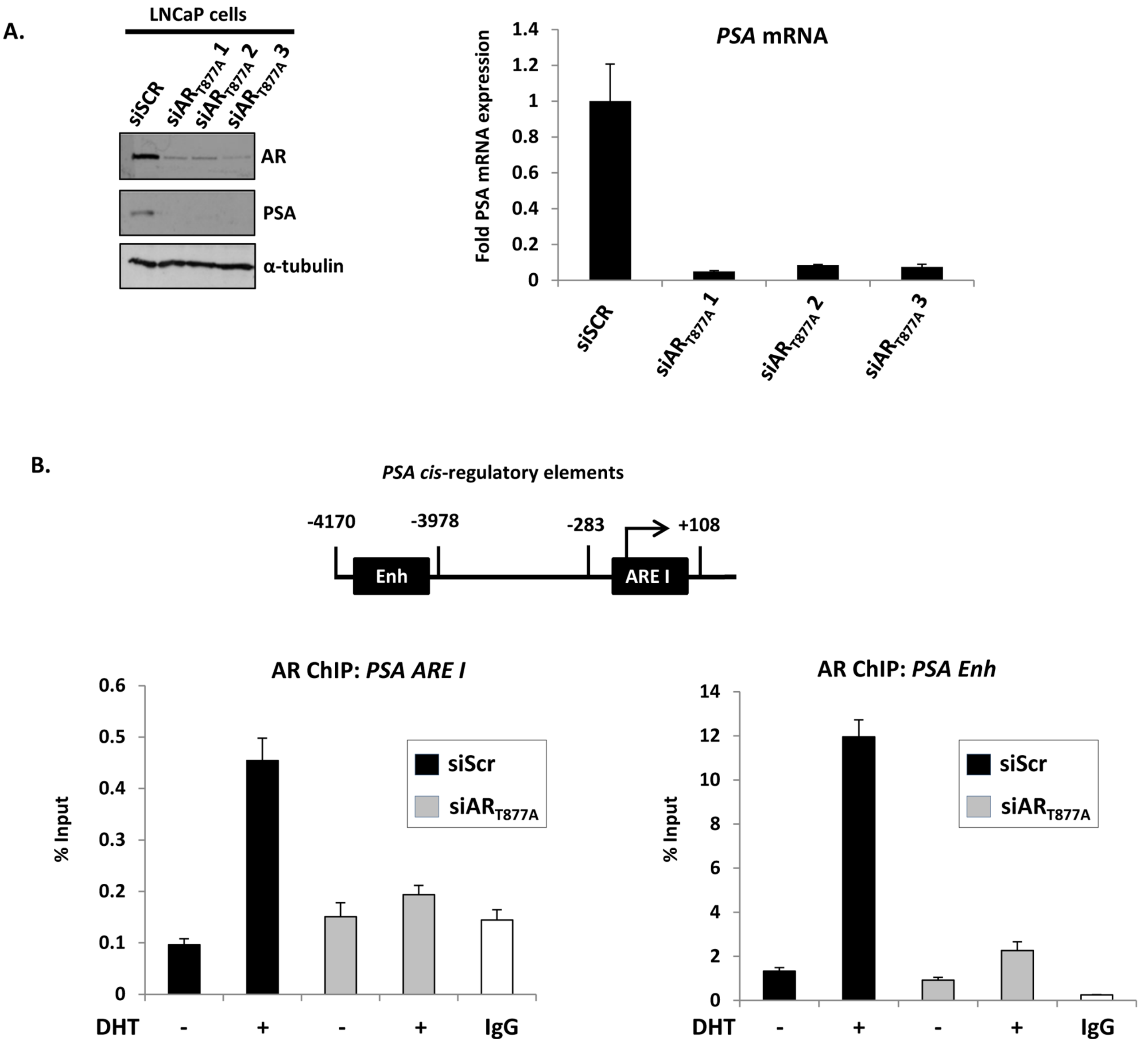

Supplementary Figure S2: AR 3′-UTR-targeting siRNAs deplete endogenous AR_{T877A} in LNCaP cells. A. LNCaP cells were transiently transfected with three AR 3′-UTR specific siRNAs (termed $siAR_{T877A}$ 1–3) or scrambled control (siScr) for 72 hours prior to western analysis using AR, PSA and α-tubulin antibodies (left panel) and quantitative PCR analysis of PSA expression (right panel). **B.** LNCaP cells depleted of endogenous AR were treated with and without 10 nM DHT for 4 hours prior to ChIP analysis using an anti-AR antibody and quantitative PCR analysis using primers specific for *PSA* promoter and enhancer elements.

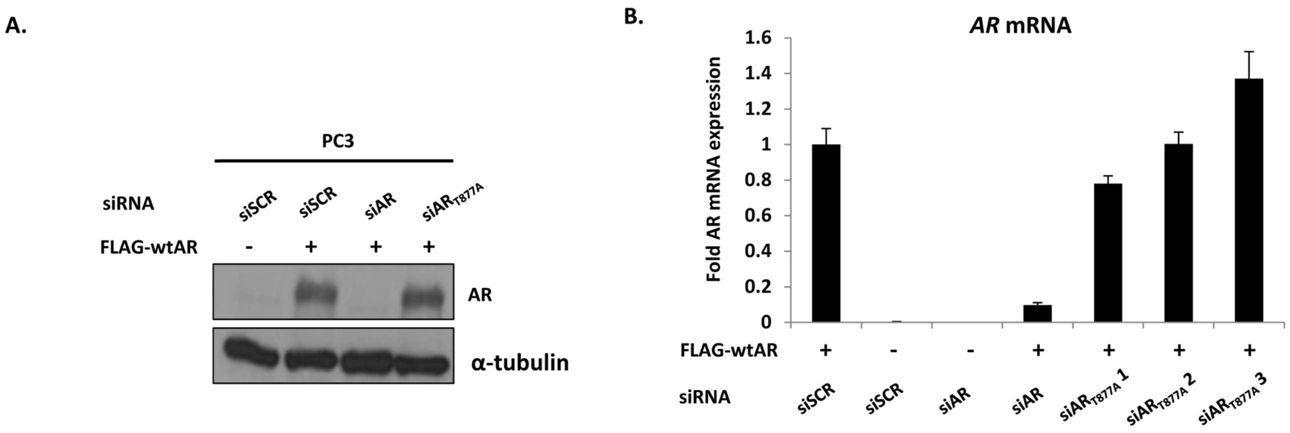

Supplementary Figure S3: Ectopically expressed AR is not targeted by AR 3′-UTR-targeting oligonucleotides. A. PC3 cells were transiently transfected with and without FLAG-tagged AR for 24 hours prior to sequential transfection with either siScr, siAR$_{T877A}$ or a coding sequence-specific AR siRNA (siAR) for 48 hours prior to western analysis using AR and α-tubulin antibodies. **B.** As in (A), but cells were subsequently harvested for RNA extraction and quantitative PCR to assess *AR* expression.

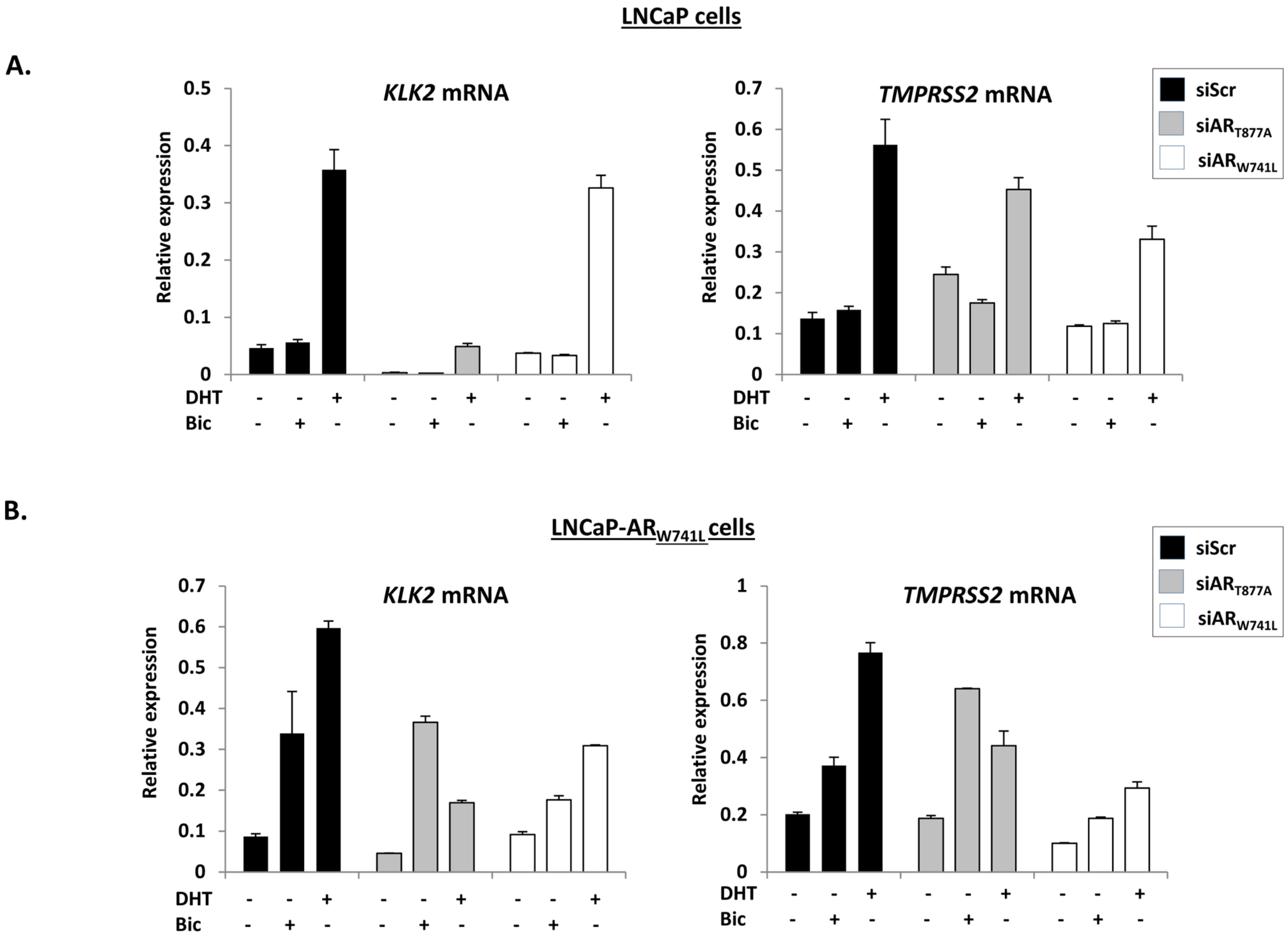

Supplementary Figure S4: Stably-integrated AR$_{W741L}$ in LNCaP cells up-regulates endogenous *KLK2* and *TMPRSS2* in the presence of bicalutamide. Quantitative PCR analysis of *KLK2* and *TMPRSS2* expression in parental LNCaP cells **A.** and the LNCaP-AR$_{W741L}$ derivative **B.** depleted of either endogenous or ectopic receptors treated with 1 nM DHT or 10 nM bicalutamide for 24 hours. Data represents the mean of three independent experiments ± standard error.

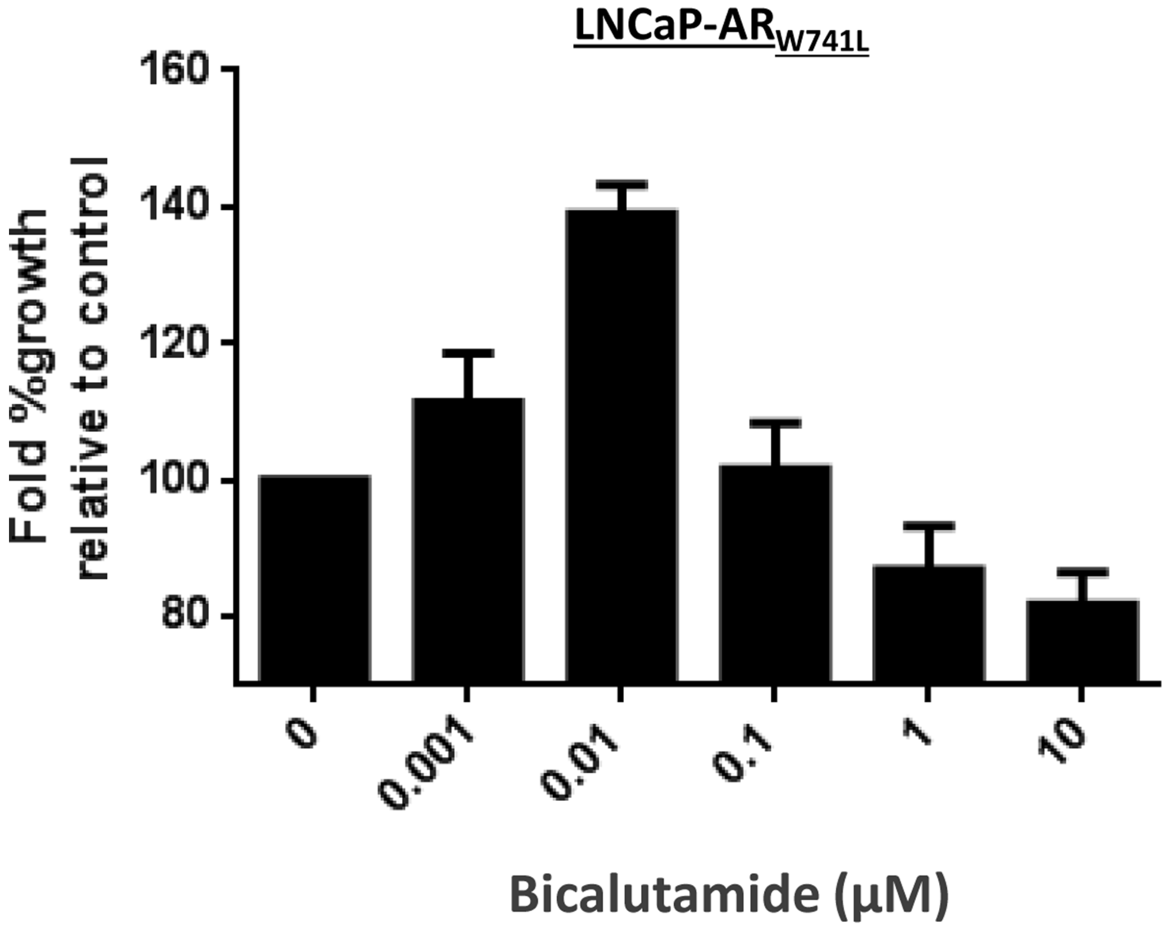

Supplementary Figure S5: Optimising bicalutamide-driven LNCaP-AR$_{W741L}$ growth. LNCaP-AR$_{W741L}$ were grown in the presence of increasing doses of bicalutamide for 96 hours prior to SRB staining.

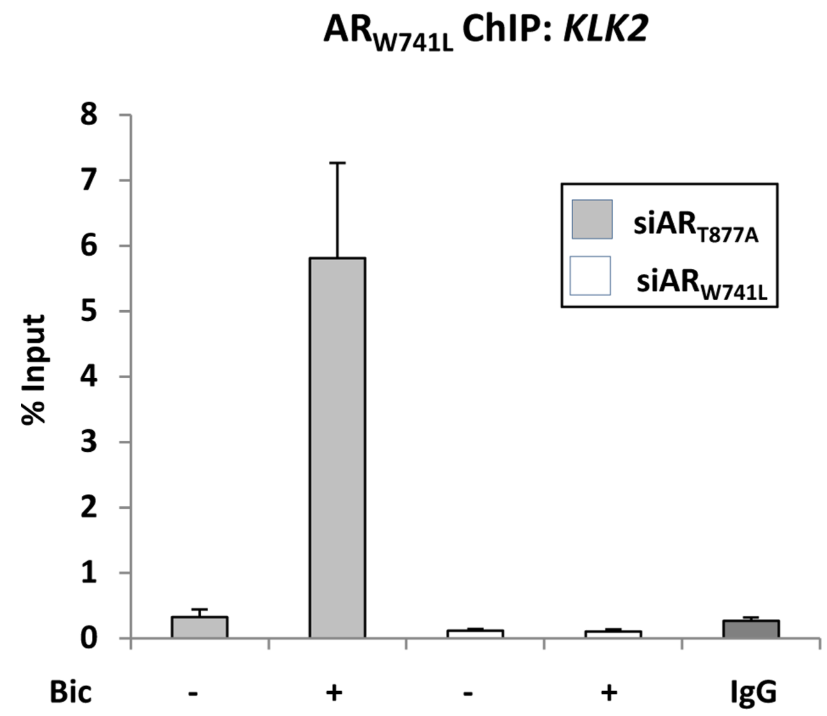

Supplementary Figure S6: AR_{W741L} is recruited to the *KLK2* promoter in response to bicalutamide. LNCaP-AR_{W741L} cells depleted of either endogenous or ectopic AR were treated with 1 nM DHT or 10 nM bicalutamide for 4 hours prior to chromatin immunoprecipitation (ChIP) analysis using a FLAG antibody to immunoprecipitate FLAG-AR_{W741L}. Receptor recruitment to the *KLK2* promoter was assessed by quantitative PCR.

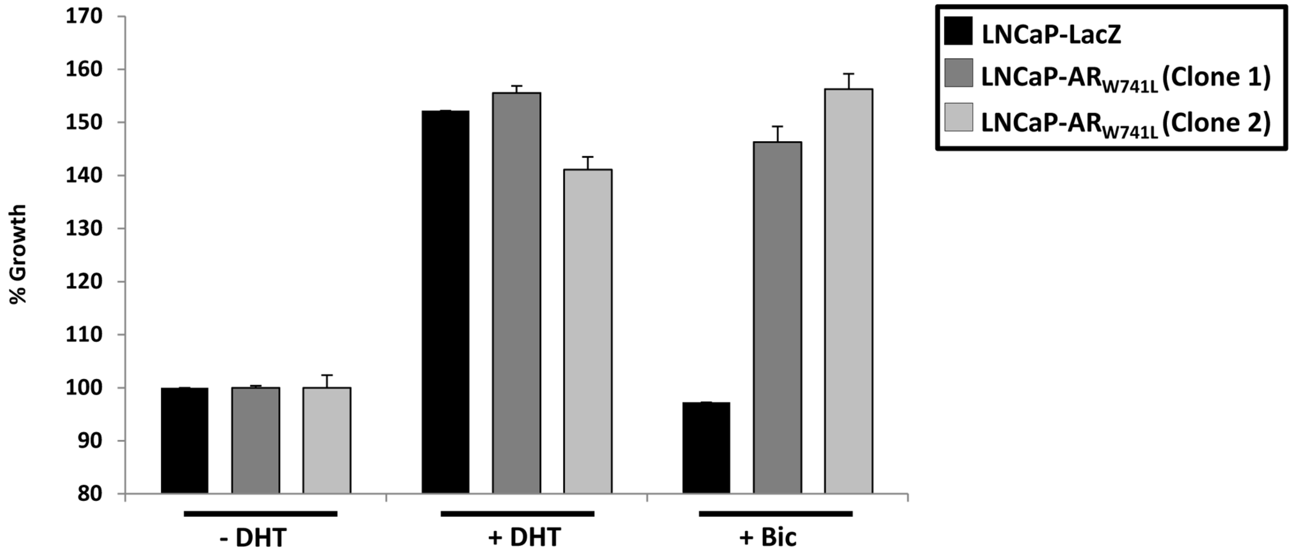

Supplementary Figure S7: Additional LNCaP-AR_{W741L} clone demonstrates equivalent growth response to bicalutamide. The growth of a second LNCaP-AR_{W741L} clonal population (LNCaP-AR_{W741L} clone 2) was tested in response to 1 nM DHT and 10 nM bicalutamide and compared to LNCaP-LacZ and the original LNCaP-AR_{W741L} clone (clone 1). After 96 hours, cells were subject to SRB staining. Data is represented as fold % growth relative to vehicle ± standard deviation.

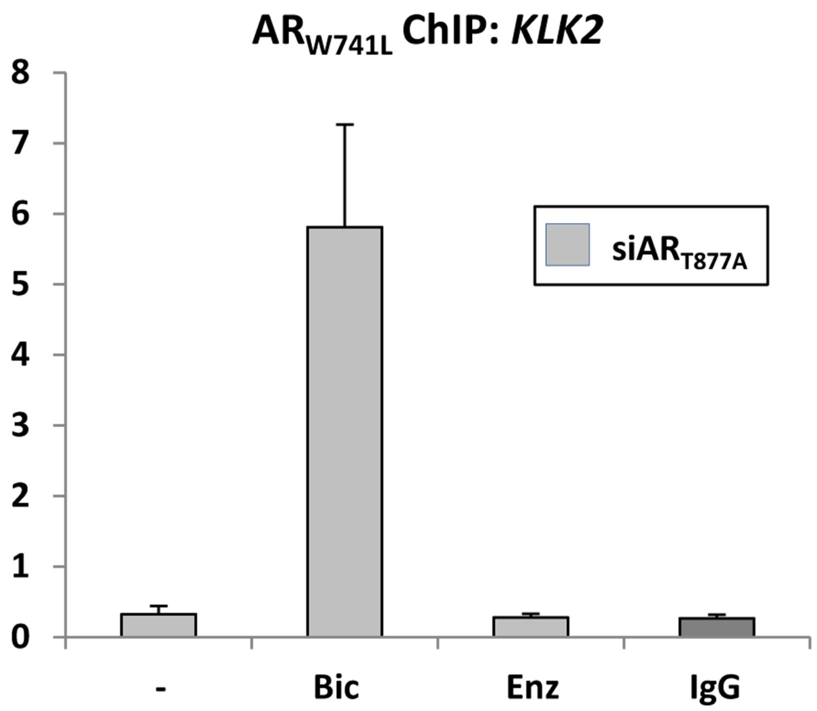

Supplementary Figure S8: Bicalutamide-driven AR_{W741L} binding to the *KLK2* promoter is attenuated by enzalutamide. LNCaP-AR_{W741L} cells transfected with siScr or $siAR_{T877A}$ were treated for 4 hours with either 1 nM DHT or bicalutamide + 10 µM enzalutamide prior to ChIP and quantitative PCR analysis using primers specific to AR-target genes.

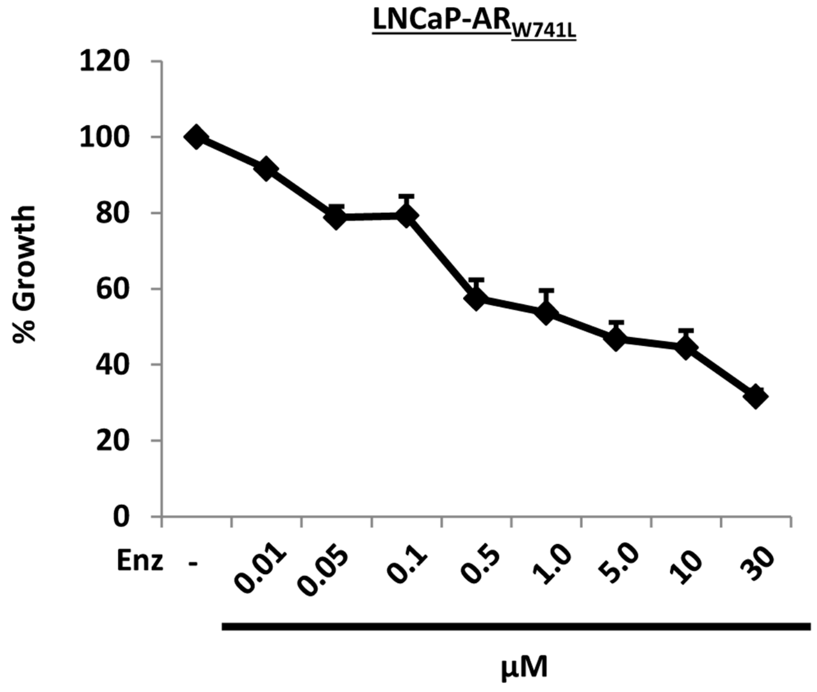

Supplementary Figure S9: Enzalutamide inhibits steady-state growth of LNCaP-AR_{W741L}. LNCaP-AR_{W741L} cells depleted of endogenous receptor using $siAR_{T877A}$ oligonucleotides were treated with an increasing dose of enzalutamide for 96 hours prior to staining with SRB. Percentage growth is relative to vehicle control. Data is the mean of two independent experiments ± standard error.

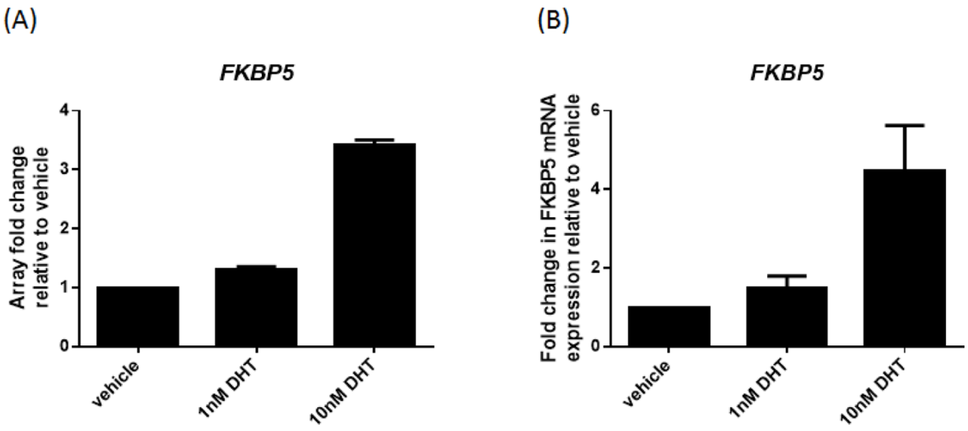

Supplementary Figure S10: DHT concentration dependent expression of FKBP5 in LNCaP-LacZ cells. Expression of FKBP5 in response to 1 nM and 10 nM DHT in LNCaP-LacZ cells was determined by **A.** bead chip array and **B.** quantitative PCR. Data are the mean of three independent experiments ± standard error.

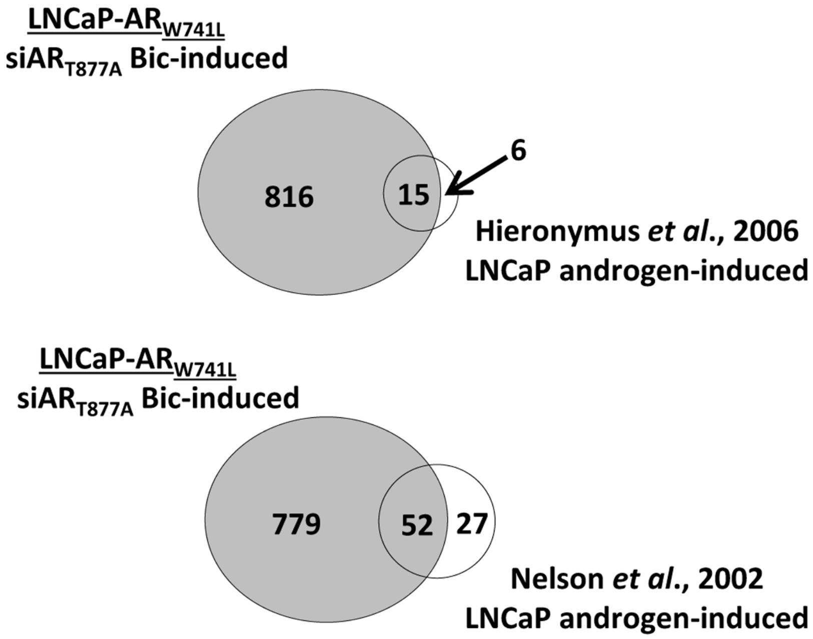

Supplementary Figure S11: Comparison of bicalutamide-activated AR_{W741L} transcriptome with defined androgenic gene signatures. The 831 core set of bicalutamide-induced genes in LNCaP-AR_{W741L} were directly compared to two androgenic signalling programs previously published by Hieronymus et al., (2006) and Nelson et al., (2002).

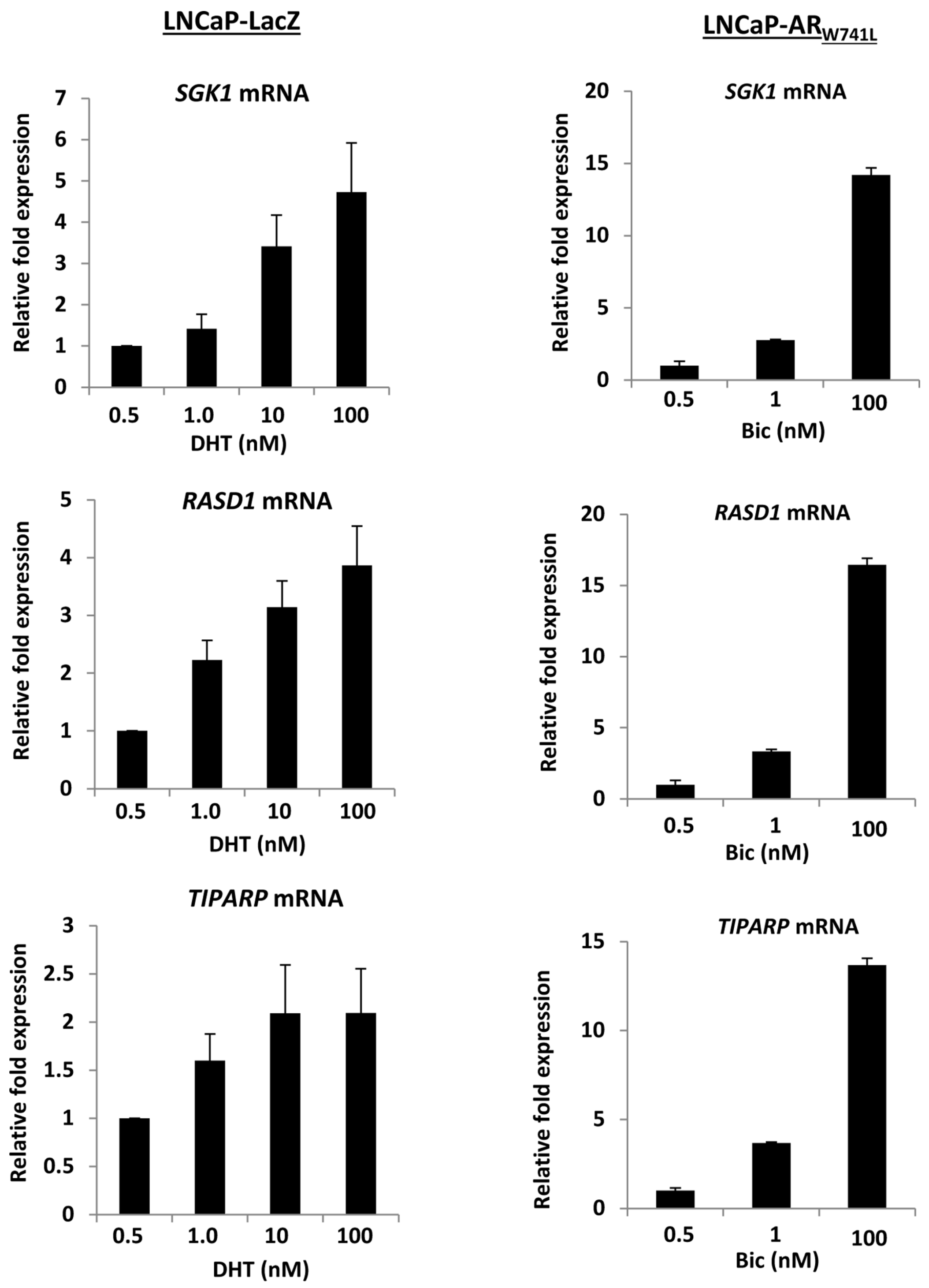

Supplementary Figure S12: *SGK1*, *TIPARP* and *RASD1* expression profiling in LNCaP-LacZ and LNCaP-AR$_{W741L}$ in response to DHT dose-range. LNCaP-LacZ cells, and LNCaP-AR$_{W741L}$ cells that were depleted of endogenous AR, were treated for 24 hours with increasing doses of either DHT or Bicalutamide (Bic), respectively, prior to quantitative analysis of *SGK1, TIPARP* and *RASD1* expression.

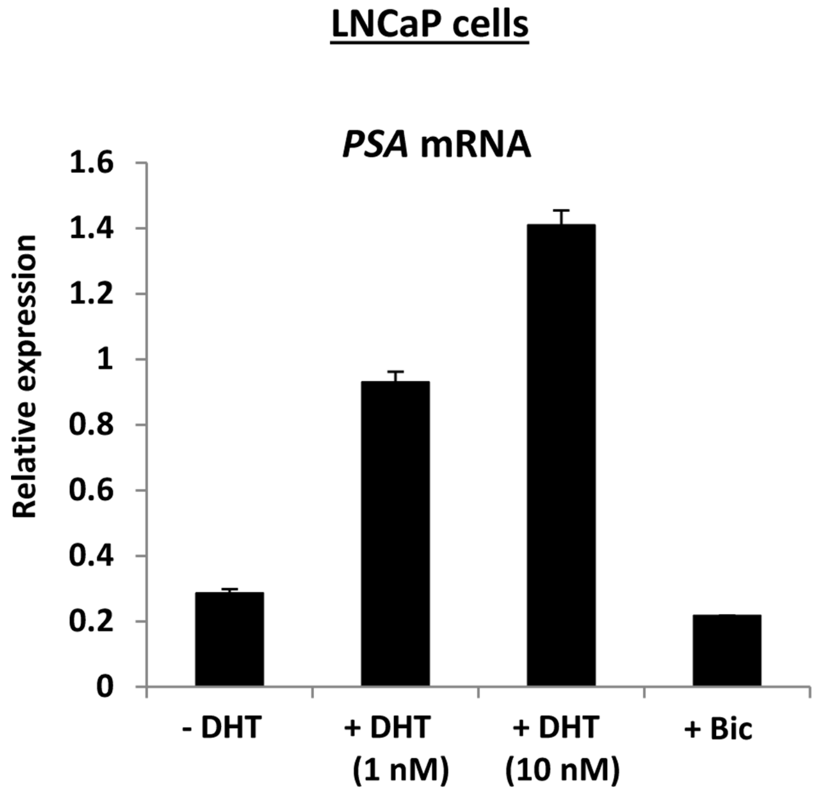

Supplementary Figure S13: Confirming response of LNCaP cells to DHT. LNCaP cells were treated for 24 hours with either 1 or 10 nM DHT prior to quantitative analysis of *PSA* expression.

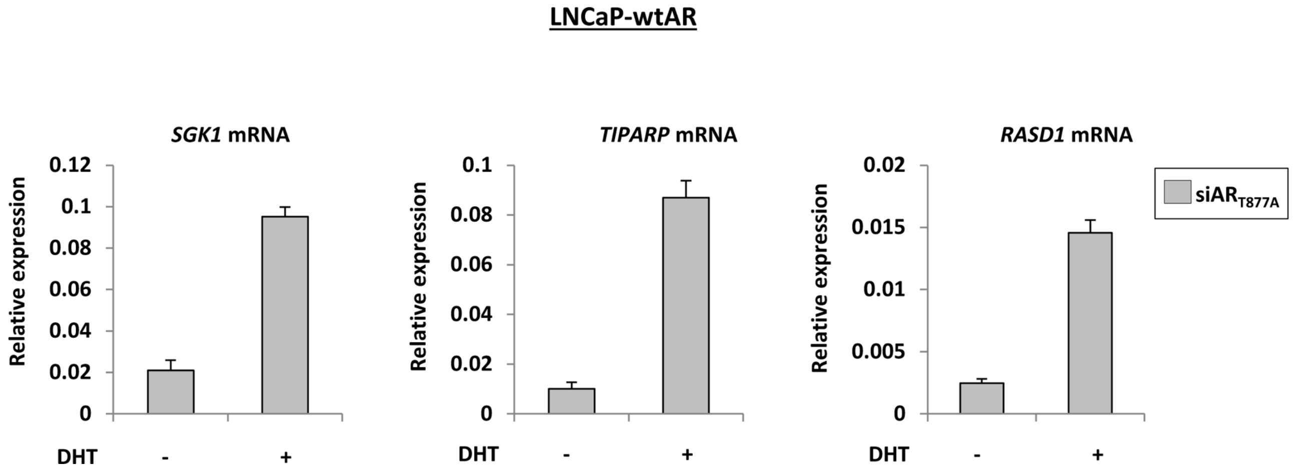

Supplementary Figure S14: *SGK1*, *TIPARP* and *RASD1* expression in LNCaP-wtAR cells. The LNCaP-wtAR cell line derivative that ectopically expresses wild-type AR was subject to endogenous AR_{T877A} knockdown for 48 hours prior to 24 hour DHT stimulation and quantitative analysis of gene expression.

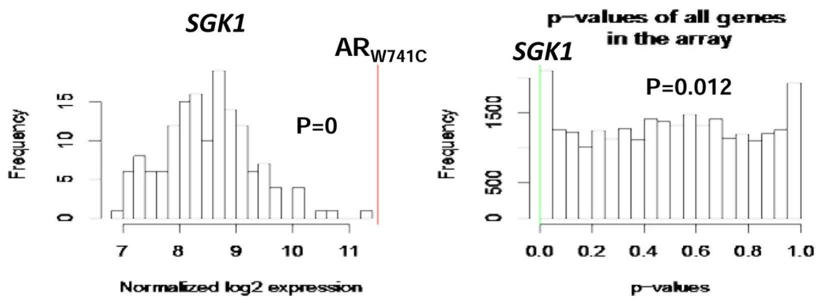

Supplementary Figure S15: *SGK1* expression is markedly up-regulated in an AR_{W741C}-expressing patient biopsy. Histograms of normalized log2 expression level of *SGK1* among the 149 non-AR_{W741C} cancer samples. Red vertical lines mark the normalized expression level of the gene in the AR_{W741C} mutant sample. To check if the *p*-value for *SGK1* is likely to be expected for other genes in the AR_{W741C} sample, genes demonstrating significantly higher expression compared to non-AR_{W741C}-expressing biopsies were identified and the probability of getting a *p*-value as extreme as observed for *SGK1* was calculated. There are 329 other genes (out of 26447) whose *p*-value is as extreme as *SGK1*; meaning the probability of getting a *p*-value as extreme as *SGK1* is 0.012 (Lower right-hand panel).

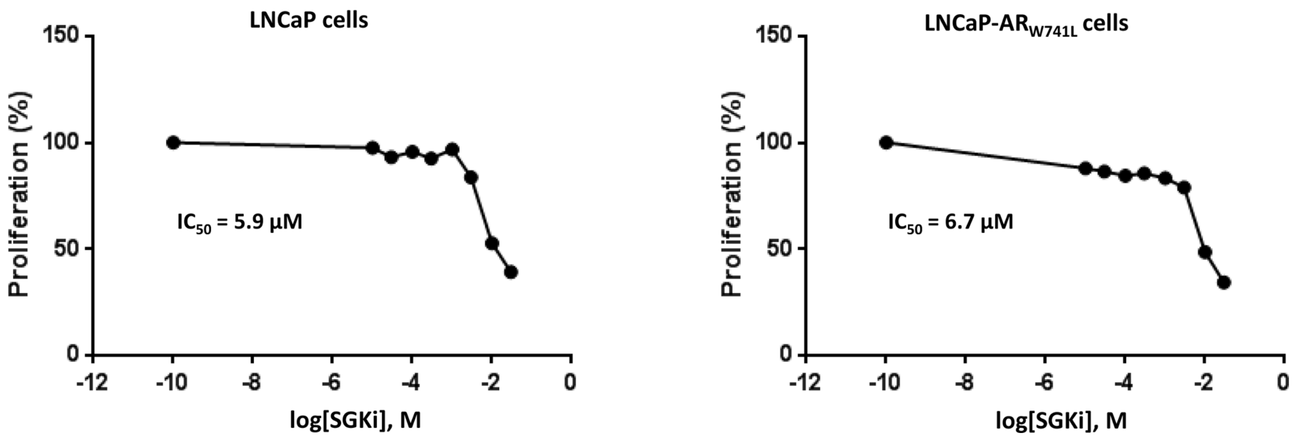

Supplementary Figure S16: IC50 values for LNCaP and LNCaP-AR_{W741L} cells. LNCaP and LNCaP-AR_{W741L} cells were grown in serum-containing conditions and treated with 0, 0.01, 0.03, 0.1, 0.3, 1, 3, 10, 30 μM SGK1 inhibitor for a total of 96 hours and cell confluency was measured using the Essenbioscience Incucyte live cell imager.

Supplementary Table S1: Sequences of forward and reverse primers used for quantitative PCR expression analysis of indicated genes and AR_{W741L} mutagenesis primers

Gene	Forward primer sequence	Reverse primer sequence
**AR**	CATGTGGAAGCTGCAAGGTCT	TCTGTTTCCCTTCAGCGGC
**PSA (KLK3)**	GGTGCATTACCGGAAGTGGAT	TGGTCATTTCCAAGGTTCCAA
**KLK2**	AGCATCGAACCAGAGGAGTTCT	TGGAGGCTCACACACCTGAAGA
**TMPRSS2**	CTGCTGGATTTCCGGGTG	TTCTGAGGTCTTCCCTTTCTCCT
**FKBP5**	CCCCCTATTTTAATCGGAGTAC	TTTTGAAGAGCACAGAACACCCT
**NDRG1**	ACAACCCCCTCTTCAACTACG	GCCAATAATGCTTTTCAGCCCA
**RASD1**	GTGTTCAGTCTGGACAACCGC	CTGCTCGATCTCGCGCTGGTC
**TIPARP**	CGTCTGGGGAGTAGGCAAT	CCCGAGGGAGGATGTGAAAC
**SGK1**	ATGACGGTGAAAACTGAGGC	GACGTGTCTTGCGGAATTTG
**HPRT1**	TTGCTTTCCTTGGTCAGGCA	AGCTTGCGACCTTGACCATCT
**PSA ARE I (ChIP)**	CCTAGATGAAGTCTCCATTGAGCTACA	GGGAGGGAGAGCTAGCACTTG
**PSA ARE III (ChIP)**	TGGGACAACTTGCAAACCTG	CCAGAGTAGGTCTGTTTTCAATCCA
**TMPRSS2 (ChIP)**	TGGTCCTGGATGATAAAAAAAGTTT	GACATACGCCCCACAACAGA
**KLK2 (ChIP)**	ACCCCTGTTGCTGTTCATCCTG	CCGCCCTTGCCCTGTTGG
**AR_{W741L} Mutagenesis**	GTCATTCAGTACTCCTTGATGGGG CTCATGGTG	
**AR_{H874Y} Mutagenesis**	CTATTGCGAGAGAGCTGTATCAG TTCACTTTTGACC	

Supplementary Table S2: Sequences of $siAR_{W741L}$, $siAR_{T877A}$ and siAR siRNA oligonucleotides

**$siAR_{T877A}$ 1**	CCAGUGUCAAGUUGUGCUUdTdT
**$siAR_{T877A}$ 2**	GCCAGCCACACAAACGUUUdTdT
**$siAR_{T877A}$ 3**	GGGAAGUUUAGAGAGCUAAdTdT
**$siAR_{W741L}$ 1**	CAGUCGACUCUAGAGGAUCCAdTdT
**$siAR_{W741L}$ 2**	UAGAGGAUCCAUGGAAGUGCAdTdT
**siAR**	CCAUCUUUCUGAAUGUCCUdTdT

Supplementary Table S3: Commercial antibodies used in study

**AR N20 (SC-816)**	Santa Cruz Biotechnology
**α-Tubulin**	Sigma
**PSA**	Gift from Kim Pettersson
**FLAG**	Sigma
**pSer5-RNA Pol II**	Abcam

Supplementary Table S4: Genes found to be up-regulated >1.5-fold in the LNCaP-AR$_{W741L}$ derivative depleted of AR$_{T877A}$ in response to bicalutamide

ABCC1	ATP6V1C1	C3orf70	CKAP2	DNAJC3	F2RL1	
ABCC4	ATP7B	C6orf106	CLDN8	DNAL1	FADS1	
ABCC8	AVEN	C6orf81	CLEC4D	DNM1L	FADS2	
ABCD3	AZGP1	C7orf54	CLGN	DNM2	FAM103A1	
ABCF3	B4GALT1	C7orf68	CLIC4	DOCK5	FAM104A	
ABHD3	BBX	C8orf42	CLPTM1L	DPH3	FAM105A	
ACAA1	BCAP29	C9orf152	CMAS	DPYSL4	FAM105B	
ACAD8	BEND4	C9orf91	CNN2	DSN1	FAM114A1	
ACAT2	BMPR1A	CALCOCO2	COL6A1	DSP	FAM129B	
ACPL2	BNIP3	CALU	COX18	DTL	FAM13AOS	
ACSL1	BRP44	CAMK1G	CPEB4	DUSP2	FAM45B	
ACSL3	C10orf114	CAMKK2	CRAMP1L	DYNC1LI1	FAM49B	
ACTA2	C10orf6	CANT1	CREB3L2	DYNLL2	FAM54A	
ADAM9	C10orf88	CAP1	CREB3L4	EAF2	FAM58A	
AGR2	C11orf82	CAP2	CREM	EBP	FAM64A	
AHNAK	C12orf23	CAPN2	CRYBB2	EDC3	FAM83F	
AK3L1	C12orf44	CAPZB	CSK	EDG7	FAM91A1	
AKAP13	C13orf1	CARD11	CTGF	EEA1	FANCF	
AKAP9	C13orf15	CARD8	CTNNAL1	EFNA1	FANCI	
ALDH1A3	C14orf118	CBLL1	CUL4B	EFNA5	FARP1	
ALG11	C14orf149	CBWD1	CWC22	EGF	FASN	
ALG2	C15orf23	CBWD3	CWF19L2	EGLN1	FBXL2	
ALKBH8	C17orf48	CBWD5	CXorf23	EGLN2	FBXL3	
AMACR	C17orf53	CCBL1	CYP2U1	EGR1	FBXO31	
ANKRD22	C17orf79	CCDC120	CYP51A1	EIF2AK3	FBXO38	
ANKRD27	C17orf80	CCDC15	CYR61	ELK4	FBXW2	
ANKRD28	C17orf91	CCDC41	DAPK3	ELL2	FCHO2	
ANKRD32	C18orf19	CD164	DBI	ELOVL5	FDFT1	
ANKRD37	C18orf54	CD9	DCBLD1	ELOVL7	FDPS	
ANLN	C19orf23	CDC14B	DDHD1	EME1	FHDC1	
ANXA2	C19orf26	CDC25A	DDX3Y	EMP1	FICD	
ANXA2P1	C19orf71	CDC45L	DEGS1	ENDOD1	FKBP5	
AP2B1	C1orf102	CDC7	DEPDC1B	ENTPD7	FKSG30	
AP3S1	C1orf116	CDH15	DHCR24	EPB41L4B	FLJ20254	
APIP	C1orf149	CDK6	DHCR7	EPDR1	FLJ20273	
ARAP2	C1orf21	CDR2	DHFR	EPS15	FLJ23754	

(*Continued*)

ARFGEF2	C1orf26	CDYL2	DHODH	ERCC6L	FLJ31568	
ARHGAP28	C1orf27	CEMP1	DIS3L2	ERGIC2	FLJ40504	
ARIH1	C1orf9	CENPC1	DISP1	ERO1L	FLJ44635	
ARMET	C20orf175	CENPN	dJ341D10.1	ERRFI1	FLNA	
ARRDC1	C21orf128	CENPO	DLL1	ESCO1	FMO4	
ARSG	C21orf56	CHAC2	DMXL1	ESCO2	FNBP1L	
ASB7	C2orf30	CHD1L	DNAJB14	ETAA1	FNDC3B	
ASF1B	C2orf49	CHEK1	DNAJB4	EVL	FOXC1	
ATAD2	C2orf76	CHST2	DNAJB9	EXOC8	FOXD4	
ATP1A1	C3orf25	CHSY1	DNAJC2	EXOD1	FOXD4L1	
FOXD4L2	HARBI1	ITPKA	LYSMD3	NAGK	PCYT2	
FOXO1	HBA2	ITPKC	LZTFL1	NAMPT	PDCD10	
FRAG1	HBEGF	ITPRIP	MALT1	NANOS1	PDE8B	
FRAP1	HCFC2	JHDM1D	MAP1LC3B	NANS	PDIA5	
FRK	HERC3	KCNG3	MAP2K4	NARG1L	PDLIM1	
FRMD3	HERPUD1	KCNK9	MAP7	NARG2	PDLIM5	
FRYL	HGD	KCNN2	MAP7D1	NCAPD3	PDS5B	
FUT11	HIF1A	KCNRG	MAP9	NCKAP1	PDXP	
FUT4	HIPK2	KCTD3	MAPK7	NDFIP2	PEA15	
FZD1	HIST1H3D	KDELR2	MASTL	NDRG1	PEBP4	
GABPB1	HIST1H4E	KDELR3	MCCC2	NEBL	PECI	
GADD45B	HIST1H4H	KDSR	MCEE	NEURL1B	PELO	
GADD45G	HJURP	KIAA0232	MCM10	NFIB	PFKFB2	
GALK2	HK2	KIAA1009	MCPH1	NFKBIA	PFKFB4	
GALNTL4	HLA-DMA	KIAA1712	MEAF6	NKX3-1	PGBD3	
GBA2	HLA-DRB4	KIAA2010	MED31	NOTCH2NL	PGC	
GBE1	HMGCR	KIF15	MERTK	NRAS	PGM3	
GBF1	HMGCS1	KIF22	MFSD6	NRG4	PHACTR2	
GCAT	HOMER1	KIFC1	MGAT2	NSDHL	PHLDA1	
GFM1	HOMER2	KLF5	MICAL1	NSMAF	PIAS1	
GFPT1	HOOK3	KLF6	MICALCL	NT5DC3	PICALM	
GINS2	HS1BP3	KLHDC8B	MICALL1	NUDT16	PIK3C2A	
GINS3	HS3ST1	KLHL2	MINPP1	NUDT18	PIP5K2A	
GK5	HS6ST1	KLHL36	MIPEP	NUDT9	PKP2	
GLB1L2	HSD17B11	KLK2	MIR342	NUP98	PMEPA1	
GLRX2	HSD3B1	KLK3	MIR611	NUPL1	PNMA1	
GLUD1	HSPA13	KLK4	MLF1IP	ODC1	PNPLA4	
GMPPA	HSPC159	KRT18	MLH3	OFD1	PNPLA8	

(Continued)

GMPPB	HTR7P	KRT8	MLPH	OGFRL1	POL3S	
GNA12	IBTK	L3MBTL3	MMAA	OKL38	POLE2	
GNAI3	ID2	LAMC1	MOBKL1B	ORMDL2	POLR3E	
GOLGA4	IDI1	LCOR	MOCOS	OSBPL11	POP1	
GOLGA5	IDO2	LDLR	MON1B	OSBPL1A	POTEE	
GOLPH3	IFFO2	LEPREL1	MORC4	OSBPL8	PPAP2A	
GOLT1B	IFRG15	LIFR	MOS	OSTC	PPFIBP1	
GOSR2	IGSF5	LIN7B	MPHOSPH10	OSTCL	PPIB	
GPR137C	IKBIP	LMTK2	MPHOSPH9	P4HA1	PPM1A	
GPR37	IL6R	LONRF1	MPZL1	PACS1	PPM1B	
GPR89B	IL6ST	LPAR3	MRFAP1	PACSIN1	PPM1D	
GPX8	IMMP1L	LPIN1	MRPS35	PACSIN2	PPM2C	
GRHL1	IMPDH1	LRIG1	MSX1	PAFAH1B2	PPP1CB	
GSTT2	INPP4B	LRRC16	MTMR6	PAK1IP1	PPP1R15B	
GSTT2B	INTS10	LRRC16A	MTMR9	PARP16	PPP2CB	
GTSE1	IQCB1	LRRC3	MTP18	PARVA	PPP3CC	
H2AFV	IRS2	LRRC59	MYL12A	PAX7	PPP4R2	
H2AFZ	ITGAV	LRRC8A	MYO9B	PCTP	PPPDE1	
PQLC1	RNU6ATAC	SLC30A1	STAMBPL1	TPM1	WDR33	ZNF695
PRAGMIN	RP5-1022P6.2	SLC31A2	STARD4	TRAPPC2P1	WDR41	ZNF761
PRDX6	RPRC1	SLC33A1	STAU2	TRIM24	WDR55	ZNF844
PREB	RPRD1B	SLC35F2	STIL	TRIM36	WDYHV1	ZSCAN12L1
PRKAB2	RPS6KA6	SLC38A1	STK35	TRIM48	WIPI1	
PRKCABP	RRAS	SLC38A2	STK39	TRIM52	WNK3	
PSMD8	RRAS2	SLC39A1	STOML1	TRIO	WNT7B	
PTGER4	RRBP1	SLC39A10	STYK1	TRQ1	WSB1	
PTGFR	RREB1	SLC39A7	SURF4	TSC22D1	WWC1	
PTPRM	RRM2	SLC41A1	SYNJ1	TSEN15	YTHDF1	
PUSL1	RSU1	SLC45A3	TAF1A	TSKU	YWHAQ	
PVRL3	S100P	SLC7A11	TARP	T-SP1	ZBED3	
PWP1	S1PR4	SLCO2A1	TARS	TSPYL2	ZBTB10	
PYCRL	SACS	SMAP1	TASP1	TTC30B	ZBTB16	
PYGB	SAMD8	SMPD2	TBC1D15	TTK	ZBTB24	
PYROXD1	SAP30	SMS	TBC1D16	TUBA3D	ZBTB25	
RAB11FIP1	SASH1	SNAPC5	TBC1D4	TUBA3E	ZBTB8A	
RAB32	SAT1	SNORA4	TBC1D8	TUBB2A	ZC3H12A	
RAB3B	SC4MOL	SNORA59B	TBRG1	TUFT1	ZCCHC6	
RAB4A	SCAP	SNORA80	TCEAL3	TXNDC11	ZCCHC9	

(Continued)

RAD54L	SCARNA11	SNORD88B	TEDDM1	TXNL4B	ZDHHC20	
RALBP1	SCNN1G	SNTB2	TEX2	UAP1	ZDHHC21	
RALGPS2	SEC13	SNX10	TGFBR2	UBE2G1	ZDHHC8P	
RALY	SEC14L1	SNX22	THEX1	UBE2J1	ZDHHC9	
RANBP2	SEC24A	SNX25	THYN1	UBE2NL	ZFHX3	
RANBP2L1	SEC24D	SOAT1	TIPARP	UCK2	ZFP36	
RAP1GAP	SELS	SOCS2	TMED7	UGDH	ZNF101	
RASA3	SEPP1	SPATA13	TMEM127	UHRF2	ZNF18	
RASD1	SERP1	SPATA2	TMEM149	UPP1	ZNF185	
RASSF3	SGK	SPC25	TMEM150A	USO1	ZNF215	
RBM28	SGK1	SPCS3	TMEM164	USP10	ZNF226	
RBM45	SGMS2	SPDEF	TMEM2	USP30	ZNF259	
RBPJ	SGOL1	SPDYA	TMEM214	USP33	ZNF286C	
RCAN1	SGSM1	SPHAR	TMEM39A	USP38	ZNF347	
RCBTB1	SHRM	SPOCK1	TMEM56	USP48	ZNF350	
RER1	SHROOM3	SPRED2	TMEM79	USP6	ZNF367	
RFPL1	SIK1	SPRYD5	TMEM8	USP8	ZNF470	
RGMB	SIPA1L2	SPSB1	TMEM87B	VCL	ZNF518B	
RGPD1	SKIL	SQLE	TMPRSS2	VDR	ZNF542	
RGPD2	SLBP	SRD5A1	TNFAIP3	VGF	ZNF552	
RGS9BP	SLC16A6	SRP19	TNFRSF10A	VPS26B	ZNF57	
RHOB	SLC25A13	SSR1	TNFRSF10B	VPS33B	ZNF570	
RHOBTB2	SLC25A30	SSR2	TNFRSF12A	VPS37B	ZNF597	
RHOU	SLC26A2	SSR3	TOR1AIP2	VPS4A	ZNF611	
RIMS4	SLC29A2	SSX2IP	TOR2A	WDFY2	ZNF613	
RIT1	SLC2A1	ST3GAL4	TP53I11	WDR1	ZNF695	

Supplementary Table S5: 1 nM DHT-activated genes in the LNCaP-LacZ derivative. Genes highlighted in green are also up-regulated in response to 10 nM DHT

ABCC4	HMOX2	SNORD30
ALDH1A3	HOMER2	SNORD46
AMAC1L2	IL1RAPL1	SNORD88B
APOA1	INPP4B	SPPL2B
ASF1B	IPPK	TARP
AZGP1	KCNN2	TBC1D25
BTG1	KLK2	TBX19
C12orf77	KLK3	TMEFF2
C16orf75	KLK4	TMEM79
C19orf48	LCP1	TMPRSS2
C1orf116	LIG4	TP53TG1
CC2D2A	LIN9	TRIM65
CCDC74A	LSP1	TRPM8
CDC45L	MAD2L1	TUBA3D
CDCA5	MCCC2	TUBB2A
CDKN2B	MCM4	UBE2K
CDT1	MED13	VGF
CENPK	MEG8	VRK1
CENPN	MESP1	WAPAL
CLDN8	MTHFD1L	ZBTB16
CLGN	MTMR12	
CROT	MTP18	
CRYM	MUC2	
DHCR24	NCAPG	
DHODH	NKX3-1	
DLG2	PALMD	
E2F2	PASK	
EAF2	PDIA5	
EDG7	PLA2G7	
ELOVL5	PMEPA1	
FAM101B	PPP1R15B	
FAM174B	PRAGMIN	
FAM49B	PRINS	
FAM64A	PSMC3IP	
FANCB	RCN3	
FANCI	RNF150	

(*Continued*)

FEN1	RNU86	
FERMT1	RRP9	
GINS2	SAC	
GINS3	SAP30	
GINS4	SCARNA9L	
GLRX2	SGEF	
GREB1	SLC29A1	
GTDC1	SLC45A3	
HELLS	SNORD11	

Supplementary Table S6: 10 nM DHT-activated genes in the LNCaP-LacZ derivative

ABCC4	C13orf1	CDT1	FADS1	GSTT2	LIMCH1
ACOX3	C13orf7	CENPK	FAM105A	GTDC1	LIN7B
ACSL3	C14orf106	CENPM	FAM105B	GTF3A	LIN9
ACSM3	C15orf23	CENPN	FAM110B	GUCY1A3	LMNB1
ACTR3	C15orf29	CHRNA2	FAM129A	HAUS8	LONRF1
ADI1	C16orf61	CIDEB	FAM174B	HEBP2	LPAR3
ADRA2A	C16orf75	CKS1B	FAM176B	HERC3	LPXN
AGPAT5	C17orf48	CLDN8	FAM188B	HERC5	LRP8
AGR2	C17orf98	CLGN	FAM46D	HGD	LSM6
ALDH1A3	C19orf10	CMTM7	FAM49B	HLA-DRA	LYAR
AMAC1L2	C19orf23	COG5	FAM55C	HMGB1	MAD2L1
AMHR2	C19orf36	CREB3L2	FAM65B	HMGB2	MAK16
ANKRD22	C19orf48	CREB3L4	FANCI	HMOX2	MAP3K14
ANKRD37	C1orf116	CRELD2	FBXO5	HOMER1	MAP3K5
APBB1IP	C1orf201	CROT	FEN1	HOMER2	MAP9
APIP	C1orf21	CRYM	FERMT1	HSPA5	MCCC2
APLN	C1orf24	CUTC	FERMT3	HSPC111	MCEE
APOD	C21orf56	CYR61	FICD	HUS1B	MCM2
ARFIP2	C2orf76	DBI	FKBP5	ICAM3	MCM4
ARG2	C3orf15	DEGS1	FLJ12684	IDH1	MCM5
ARID3A	C3orf25	DHCR24	FLJ23754	IDI1	MCTS1
ARMET	C6orf173	DHRS3	FLJ39827	IER3IP1	MED30
ARSG	C7orf54	DLG2	FOXD4	IL1RAPL1	MEIS1
ASAH1	C9orf140	DNAJB9	FOXD4L1	IMPA2	MESP1
ASF1B	C9orf152	DNAJC25	FOXD4L2	INHBB	MEX3B
ASNA1	C9orf72	DNASE1	FRAP1	INPP4B	MGC29506

(*Continued*)

ASPM	C9orf91	DNM1L	FSIP1	INSIG1	MGC35440
ATAD2	C9orf98	DOCK2	GAGE12D	IQGAP2	MICAL1
ATMIN	CALHM2	DYNLRB2	GAL	IRS2	MIR1974
ATP11B	CBWD3	E2F1	GALK2	ITGAV	MMEL1
AZGP1	CBWD5	E2F2	GAS1	JPH1	MORC4
B2M	CCAR1	EAF2	GDF15	KCNN2	MPHOSPH9
B3GNTL1	CCDC74A	EBP	GGCT	KDELR2	MPP6
B4GALT1	CCDC74B	EDG7	GINS2	KIAA1244	MPZL2
BEND4	CCDC86	EIF2S1	GINS3	KIF11	MT1X
BET1	CCNA2	EIF4E1B	GINS4	KIF22	MTHFD1L
BHLHB2	CCNB2	ELK4	GLB1L2	KLF15	MTMR9
BMPR1A	CCRN4L	ELL2	GLIPR2	KLK2	MTP18
BMPR2	CD320	ELOVL1	GLRX2	KLK3	NANS
BRCA1	CDC25A	ELOVL5	GLUD1	KLK4	NCAPD3
BRP44	CDC45L	EPDR1	GLYATL1	KRT18	NCAPG
BTG1	CDCA5	ERGIC2	GMFG	L3MBTL3	NDRG1
BTN3A3	CDCA7	ERP44	GNB4	LAMC1	NDUFA5
C11orf82	CDH15	ERRFI1	GPM6B	LCP1	NEDD4L
C12orf73	CDK2AP2	EXOSC5	GREB1	LDLR	NEFH
C12orf77	CDK5R2	EXTL2	GRHL2	LGALS1	NFKBIA
NKX3-1	PPAPDC1B	SAP30	SNORD56	TWIST1	
NOP16	PPFIBP1	SASH1	SNORD57	TWISTNB	
NOSTRIN	PPFIBP2	SCARNA9	SNORD73A	UAP1	
NPHP4	PPIB	SDF2L1	SNORD88B	UBE2J1	
NR2C2AP	PRAGMIN	SEC11C	SNORD99	UBE2K	
NSMAF	PRDX6	SEC24A	SOAT1	UCHL5	
NUBP1	PRKCA	SEC24D	SPATA13	UCHL5IP	
NUDT16P	PRRT2	SEC61B	SPCS3	UGT2B28	
NUP35	PSMC3IP	SEMA3C	SPDEF	UHRF2	
NUP62CL	PTGER4	Sep-07	SPHAR	UNC84B	
NXT1	PTMA	SERPINE2	SRM	VGF	
OAT	PTPRM	SFXN2	SSR3	VN1R1	
OAZ3	PTRH2	SGEF	SSSCA1	VRK1	
OR7E91P	PUSL1	SH2D1A	STARD3NL	WAS	
OSBPL5	PXDN	SLBP	STK39	WDYHV1	
OSTCL	PYCRL	SLC16A6	STRA13	WIPI1	
P2RY5	QPCTL	SLC1A5	SYTL1	WWC1	

(Continued)

PAK1IP1	RAB27A	SLC22A4	TAF1A	WWOX	
PALMD	RAB32	SLC25A15	TARP	ZBTB10	
PAQR4	RAB33B	SLC29A1	TBC1D4	ZBTB16	
PARVA	RAB36	SLC2A12	TFAP4	ZCCHC6	
PASK	RAB3B	SLC30A7	TIMP1	ZDHHC23	
PCNA	RAB4A	SLC35F2	TMEFF2	ZFP14	
PCTP	RAD51AP1	SLC36A1	TMEM109	ZNF124	
PCYT2	RANBP2	SLC39A10	TMEM149	ZNF215	
PDIA4	RAP1A	SLC39A6	TMEM194	ZNF285A	
PDIA5	RARB	SLC41A1	TMEM231	ZNF350	
PDLIM5	RBMY2FP	SLC43A1	TMEM39A	ZNF556	
PDSS1	RER1	SLC45A3	TMEM52	ZNF670	
PEA15	RFP	SLC9A2	TMEM55A	ZNF714	
PFKFB2	RFPL2	SMAD9	TMEM79	ZNF844	
PGM2	RFPL3	SMPD2	TMPRSS2		
PGM3	RHOBTB3	SMS	TMSB15A		
PHF11	RHOU	SND1	TNFAIP8		
PIGH	RIMS4	SNHG1	TOP2A		
PIK3AP1	RLN1	SNORA24	TP53AP1		
PLA2G7	RNF150	SNORA32	TP53TG1		
PLD4	RNU4-2	SNORA4	TRAM1L1		
PMAIP1	RNU86	SNORA41	TRIM48		
PMEPA1	RPIA	SNORA57	TRIM61		
PMM2	RPL22L1	SNORD11	TRIP13		
PMS1	RPS7	SNORD12C	TRPM8		
PNKD	RRP9	SNORD16	TRQ1		
POLR3K	S100A10	SNORD30	TSPAN1		
POP7	S100A11	SNORD31	TTK		
PPAP2A	S100A6	SNORD32A	TUBA3D		

Supplementary Table S7: Overlap of AR_{W741L} bicalutamide-activated transcriptome with known AR-target gene signatures: Hieronymous *et al.*, 2006 (left panel) and Nelson *et al.*, 2002 (right panel)

Hieronymus *et al.*, (2006) Gene expression signature	LNCaP-AR_{W741L} + 10 nM casodex	LNCaP LacZ + 10 nM DHT
KLK3		
TMPRSS2		
NKX3.1		
KLK2		
GNMT		
PMEPA1		
MPHOS9		
ZBTB10		
EAF2		
BM039		
SARG		
ACSL3		
PTGER4		
ABCC4		
NNMT		
ADAM7		
FKBP5		
ELL2		
MED28		
HERC3		
MAF		

Nelson *et al.*, (2002) Gene expression signature	LNCaP-AR_{W741L} + 10 nM casodex	LNCaP LacZ + 10 nM DHT
ABCC4		
ACTN1		
ADAMTS1		
AKAP12		
AKAP2		
ALDH1A3		
ANKH		
APPBP2		
AZGP1		
B2M		
B4GALT1		

**Nelson *et al.*, (2002) Gene expression signature**	**LNCaP-AR$_{W741L}$ + 10 nM casodex**	**LNCaP LacZ + 10 nM DHT**
CAMKK2		
CDC14A		
CPD		
DBI		
DCTN3		
DHCR24		
DNAJB9		
ELL2		
ELOVL5		
FACL3		
FAD55		
GP110		
GSR		
GUCY1A3		
H1F0		
HERC3		
HMGCS1		
HOMER2		
ID2		
IDI1		
INPP4B		
IQGAP2		
ITGAV		
KLF4		
KLK2		
KLK3		
KLK4		
KRT18		
KRT19		
KRT8		
LIFR		
LMAN1		
MAF		
MERTK		
MLCB		
MRF2		
NDRG1		
NKX3.1		

Nelson *et al.*, (2002) Gene expression signature	LNCaP-AR$_{W741L}$ + 10 nM casodex	LNCaP LacZ + 10 nM DHT
ORM1		
PDLIM5		
PGM3		
PHYH		
PIAS1		
PPAP2A		
PTPN21		
RAB4		
SAT		
SEC24D		
SEPP1		
SGK		
SLC26A2		
SMS		
SNAP25		
SORD		
SPAK		
SPDEF		
SPRP19		
TCRG/TARP		
TMEPA1		
TMPRSS2		
TNFAIP8		
TPD52		
TSC22		
UAP1		
UGHD		
UNC13		
VAPA		
ZBTB10		
